# Conservation planning for the endemic and endangered medicinal plants under the climate change and human disturbance: a case study of *Gentiana manshurica* in China

**DOI:** 10.3389/fpls.2023.1184556

**Published:** 2023-07-26

**Authors:** Hui Zou, Bingrui Chen, Boyan Zhang, Xinyu Zhou, Xiyuan Zhang, Xinxin Zhang, Jianwei Wang

**Affiliations:** ^1^ Heilongjiang Research Center of Genuine Wild Medicinal Materials Germplasm Resources, School of Life Sciences and Technology, Harbin Normal University, Harbin, China; ^2^ College of Basic Medicine, Heilongjiang University of Chinese Medicine, Harbin, China

**Keywords:** *Gentiana manshurica*, optimized MaxEnt model, potential distribution, environmental variables, landscape pattern, habitat quality, conservation management

## Abstract

Human activities and climate change have significantly impacted the quantity and sustainable utilization of medicinal plants. *Gentiana manshurica* Kitagawa, a high-quality original species of Gentianae Radix et Rhizoma, has significant medicinal value. However, wild resources have experienced a sharp decline due to human excavation, habitat destruction, and other factors. Consequently, it has been classified as an Endangered (EN) species on the IUCN Red List and is considered a third-level national key-protected medicinal material in China. The effects of climate change on *G. manshurica* are not yet known in the context of the severe negative impacts of climate change on most species. In this study, an optimized MaxEnt model was used to predict the current and future potential distribution of *G. manshurica*. In addition, land use data in 1980, 2000, and 2020 were used to calculate habitat quality by InVEST model and landscape fragmentation by the Fragstats model. Finally, using the above-calculated results, the priority protection areas and wild tending areas of *G. manshurica* were planned in ZONATION software. The results show that the suitable area is mainly distributed in the central part of the Songnen Plain. Bio15, bio03, bio01, and clay content are the environmental variables affecting the distribution. In general, the future potential distribution is expected to show an increasing trend. However, the species is expected to become threatened as carbon emission scenarios and years increase gradually. At worst, the high suitability area is expected to disappear completely under SSP585-2090s. Combined with the t-test, this could be due to pressure from bio01. The migration trends of climate niche centroid are inconsistent and do not all move to higher latitudes under different carbon emission scenarios. Over the past 40 years, habitat quality in the current potential distribution has declined yearly, and natural habitat has gradually fragmented. Existing reserves protect only 9.52% of *G. manshurica*’s priority conservation area. To avoid extinction risk and increase the practicality of the results, we clarified the hotspot counties of priority protection area gaps and wild tending areas. These results can provide an essential reference and decision basis for effectively protecting *G. manshurica* under climate change.

## 1 Introduction

Global climate change has long been a pressing research issue, with the loss of biodiversity caused by climate change and human activities being one of the most serious problems (Mantyka-pringle et al., 2012). Understanding and predicting how species respond to global climate change is crucial in biodiversity research (Lan et al., 2022). Numerous studies have shown that the geographic distribution patterns of species change in response to the impacts of climate change (Yu et al., 2021). The rates of species change vary widely in species characteristics and variational external drivers (Chen et al., 2011). Most studies have pointed out the profound effects of global warming on species distributions. Climate and geology have shaped ecosystems and evolution in the past, while human forces may now outweigh these across most of the Earth’s land surface today (Yue et al., 2011). Landscape structures are altered, and habitats are fragmented and degraded by changing land use, so most of the natural landscape is now embedded within anthropogenic land use and land cover mosaics (Ellis and Ramankutty, 2008). Populations of species restricted to these habitats are often spatially fragmented and threatened (Opdam and Wascher, 2004). Most studies suggest that habitat loss and fragmentation now outweigh the response of species and ecosystems to climate change. However, the effects of climate change are expected to increase over time and eventually outweigh land use change in determining population trends (Mantyka-pringle et al., 2012). Overall, there is growing evidences that climate change will interact negatively with habitat loss and fragmentation and contribute synergistically to biodiversity degradation at the species, genetic, and/or habitat levels (Mantyka-pringle et al., 2012).

The niche breadth-range size hypothesis states that by utilizing a greater array of resources and maintaining viable populations within a wider variety of conditions, a species should become more widespread, leading to a positive correlation between niche breadth and geographical range size (Brown, 1984). Most studies have tested this hypothesis (Boulangeat et al., 2012; Slatyer et al., 2013). Carscadden K. A. thus inferred that species with narrow distribution ranges would further narrow their distribution ranges under changed climate in the future, which also has been tested in the majority studies (Beaumont and Hughes, 2002; Tang et al., 2021a). However, the extent to which climate limits the distribution of endemic species is unclear (Morueta-Holme et al., 2010). Human activities have reduced the ranges of narrow-ranged species but expanded those of widespread species in China, leading to rare and distinct species being losers (Xu et al., 2019). Although narrow-ranged species are known to be more vulnerable to climate change, few studies have assessed climate change impacts (Dubos et al., 2021). Therefore, to maintain sustainable use of endangered wild resources, it is necessary to characterize the ecology of their populations, investigate their current potential distribution, determine the response of species to global warming, and then identify sites for protection and conservation (Li et al., 2020; Yoo et al., 2022). Strategically, protecting the natural environment (*in situ* conservation) will be the most effective way to maintain populations and promote gene flow (Hellmann et al., 2012; Krishnan et al., 2013). However, the climatic conditions available to species in static reserves will change as climate change intensifies, and the positive effects of reserves on species conservation will decline. That human activities cause structural disconnections in protected area networks leads to habitat loss and fragmentation, exacerbating this change (Asamoah et al., 2021). Therefore, when constructing protected areas, it is necessary to consider the impact of future climate and strong human activities on species distribution (Platts et al., 2014).

*Gentiana manshurica* Kitagawa (Gentianaceae: *Gentiana*) is a perennial herb (Editorial Board of Flora of China, 2016). It has great medicinal value and is used as traditional Chinese medicine (TCM) Gentianae Radix et Rhizoma for its dried roots and rhizomes. It is widely used in Europe and Asia (Tanaka et al., 2014; Jiang et al., 2021). Among the various original species, *G. manshurica* is the mainstream of excellent-quality commercial products (Guo et al., 2001; Meng et al., 2011). In recent decades, however, wild resources have been severely damaged due to the biological characteristics and the destruction of grasslands caused by human disturbance. It has been classified as Endangered (EN) on the IUCN Red List and is the third-level national key-protected wild medicinal materials species in China. Some scholars have suggested changing it to the second level considering the exhausted wild resources (Meng et al., 2011). Undoubtedly, global warming and increased human disturbance will be more detrimental to the growth of narrow species than widespread species. However, the future survival potential of *G. manshurica*, as an endemic species of the western meadow grasslands in Northeast China, is unknown.

Therefore, we simulated the current and future potential distribution (CPD and FPD) of *G. manshurica* and analyzed its priority protection areas (PPAs) and wild tending areas (WTAs) to provide theoretical guidance for conservation. The objectives of this study were to (1) predict the CPD and FPD and analyze the changes in the potential distribution under different periods and carbon emission scenarios, (2) identify the key environmental variables affecting the distribution of the *G. manshurica* and analyze the environmental pressure on the potential distribution under the climate change, (3) evaluate the dynamic changes in the landscape pattern and habitat quality of the CPD, (4) based on the CPD, FPD, natural habitat quality, and landscape fragmentation of CPD, apply ZONATION model to analyze PPAs and WTAs for *G. manshurica*, and (5) provide a reference for the protection of other rare and endangered wild medicinal plants.

## 2 Materials and methods

### 2.1 Study area

Species identification is the key to species conservation (Trias-Blasi and Vorontsova, 2015). It records that *G. manshurica* mainly distributes in Northeast China, North China, East China, and South China, etc. (Editorial Board of Flora of China, 2016). Scholars have made in-depth studies on the *G. manshurica* distributed in southern China, pointing out that it should be *Gentiana scabra* Bge. ssp. *australis*, the southern subspecies of *G. scabra* Bge (Liu, 1981; Guo et al., 2001; Wang, 2005). As a result, *G. manshurica* is a narrow-distributed species in Northeast China.

**Figure 1:**
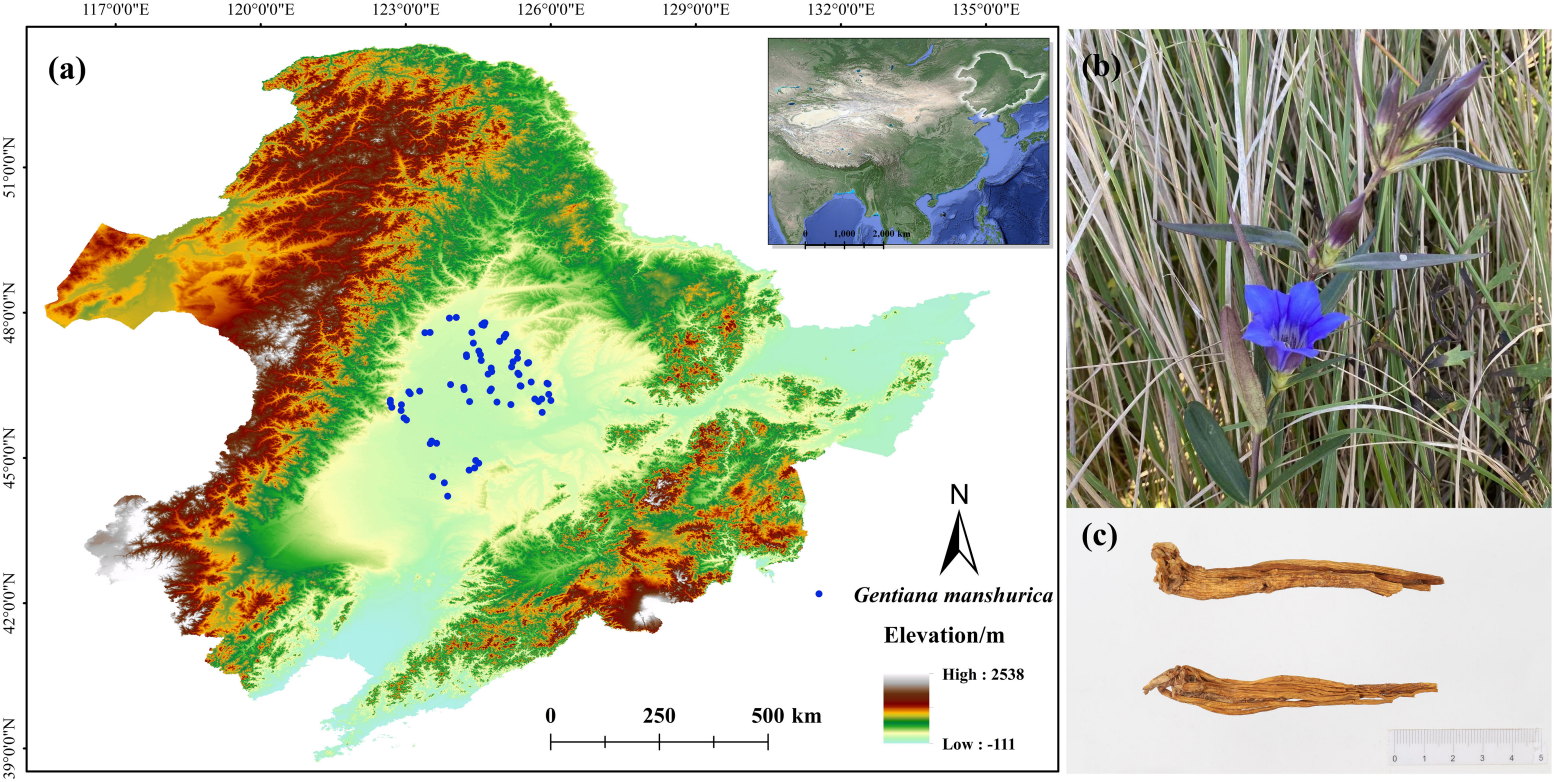
Occurrence records and morphological characteristics of *G manshurica* **(A)**. occurrence records; **(B)** flower; **(C)** dry rhizoma.

Northeast China (115°05’ ~ 135°02’ E, 38°40’ ~ 53°34’ N) is a geographical region of China, including Heilongjiang, Jilin, and Liaoning provinces, as well as Hulunbeier, Xing’an Meng, Tongliao, and Chifeng in Inner Mongolia (**Figure 1A**). The region has a complex landform, including the Greater Khingan Range, Lesser Khingan Range, Changbai Mountains, Songnen Plain, and Songliao Plain. The region features a continental monsoon climate, exhibiting chilly and arid winters alongside humid and sultry summers. And the annual average temperature of 2.75 ~ 5.72°C. From southeast to northwest, annual precipitation drops from 1,000 mm to 300 mm (Zhou and Zu, 1997).

### 2.2 Occurrence records

We obtained the occurrence records by three methods. First, 43 occurrence records were based on our field surveys from 2016 to 2022. Second, 16 occurrence records were obtained based on the Fourth Chinese Materia Medica Resources Survey. Last, 12 occurrence records were investigated based on the Chinese Virtual Herbarium (https://www.cvh.ac.cn/). All specimens were carefully examined to ensure correct identification. Invalid, duplicate, and non-natural records were removed. Then the data were spatially filtered to retain only one point in each grid cell (1×1 km) using ENMtools. Finally, we obtained 71 occurrence records of *G. manshurica*.

### 2.3 Environmental and geographic data

*G. manshurica* (**Figures 1B, C**) is mainly concentrated in the seasonal water area in the semi-humid meadow grassland area. Moreover, there are requirements for soil, such as the general distribution of salinized meadow soil, and the pH value is often greater than 8.0 (Liu, 1981). To accurately simulate the potential geographic distribution of *G. manshurica*, we comprehensively considered the habitat characteristics. Therefore, 31 environmental variables were collected to simulate the CPD, including climate and soil (**Table S1**). Soil variables were from the Dataset of soil properties for land surface modeling over China of National Tibetan Plateau Third Pole Environment Data Center with a resolution of 30” (http://data.tpdc.ac.cn/) (Dai and Shangguan, 2019). Other data were from WorldClim database with the same resolution (Version 2.1, https://worldclim.org/) (Fick and Hijmans, 2017). We used CMIP6 published by the IPCC organization for the simulation of potential distribution in the future, including four periods: 2021-2040 (2030s), 2041-2060 (2050s), 2061-2080 (2070s), and 2081-2100 (2090s). Compared to CMIP5, CMIP6 improves the ability to simulate and predict future models (Xin et al., 2020). Published to date, the global Shared Socio-economic Pathways (SSPs) represent the most comprehensive research on environmental and sustainable development. The SSPs include SSP126, SSP245, SSP370, and SSP585 (Jiang et al., 2020). It can be used for climate change research and other areas, such as biodiversity and sustainable development (Van Vuuren et al., 2017). Thus, 16 combinations of future periods and climate scenarios were used in this study. IPSL-CM6A-LR, MRI-ESM2-0, and UKESM1-0-LL were selected, and the average values of each climate variable of three general circulation models were input into the model (Brambilla et al., 2022). Since soil conditions hardly change quickly, we made these variables consistent under current and future conditions (Yu et al., 2021).

To enhance the accuracy of the model simulation while avoiding collinearity between different environmental variables, the ENMtools correlation tools were employed to analyze highly collinear variables (|r|≥0.8) (**Figure S1**) (Dormann et al., 2013; Merow et al., 2013). Using the percent contribution metric, we excluded environmental variables with a contribution rate of less than 1%, and eliminated environmental variables with low contributions among two highly collinear variables (**Table S2**). Ultimately, nine environmental variables (bio15, bio03, bio01, CL, PH, POR, TN, bio13, and bio14) were used in the MaxEnt model to predict the CPD and FPD.

The China Administrative Division Maps and nature reserves were obtained from the National Geomatics Center of China (https://www.webmap.cn/). 1980, 2000, and 2020 land use data (the resolution is 30”) and the Chinese Agricultural Natural Zoning were obtained from the Resource and Environment Science and Data Center of the Institute of Geographic Science and Natural Resource Research (https://www.resdc.cn/). MaxEnt version 3.4.4 was downloaded from the website (https://biodiversityinformatics.amnh.org/open_source/maxent/). The version of ArcGIS software used in this study was 10.5, RStudio was 3.6.3, and the ZONATION model was 4.0.0 downloaded from the website (https://www.helsinki.fi/en).

### 2.4 Prediction of the CPD and FPD

This study used the MaxEnt model to predict the CPD and FPD of *G. manshurica* in Northeast China. It was shown that the prediction results of the MaxEnt model were closely related to the feature combination (FC) and regularization multiplier (RM). And using default parameters to construct the MaxEnt model is prone to overfitting, resulting in low model transfer ability and obtaining prediction results that are not the best or even unreliable (Merow et al., 2013; Morales et al., 2017). Therefore, we used the kuenm package to adjust the FC and RM of the model to obtain the best-fitting parameter combinations with the input data. First, the package created many candidate models and evaluated and selected the best model. Candidate models were obtained by combinations of 17 regularized multiplier values (0.1 ~ 1 with interval 0.1, 2 ~ 6 with interval 1, 8, and 10) and all 31 possible combinations of feature classes (linear-L, quadratic-Q, hinge-H, product-P, and threshold-T). Second, candidate models were selected using partial ROC values to measure statistical significance, omission rates to measure the predictive power of the models, and AICc values to measure the complexity of the models. The model parameter combination with the lowest AICc value (delta.AICc=0) was selected as the best model (Cobos et al., 2019). To improve the accuracy, 75% of the occurrence records were randomly selected as the training data, and the remaining 25% as the test data. Finally, we generated 10 bootstrap replicates for each of the best calibration models with a Cloglog of the output format. Other parameters were set as default.

The prediction results were examined using the area under the curve (AUC) of the receiver operating characteristic (ROC). The closer the AUC to 1, the more accurate the model is (Tang et al., 2021b). This study used the maximum training sensitivity plus specificity Cloglog threshold (P) to determine the optimal conversion threshold, and P was 0.1899. The suitability of areas was determined based on the probability of species presence (p) being greater than or equal to P (suitable) or less than P (non-suitable) (Liu et al., 2013). Combining the treatment of uncertainties for the IPCC Fifth Assessment Report (Sun et al., 2012), the suitability area was classified as: < 0.1899 is a non-suitable area; 0.1899 ≤ p < 0.33 is low suitability area; 0.33 ≤ p < 0.66 is mid suitability area; 0.66 < p is high suitability area.

We used SDMtoolbox written in python to binarize the potential area of future climate scenarios, clarify the changes, and obtain the expansion, stability, and contraction areas (Brown et al., 2017). We could not interpret the adaptive potential of species, so our interpretation of the following results is limited to the expected changes in the distribution of environmental features associated with the presence rather than the distribution of *G. manshurica*.

Finally, to assess the novelty of future climate conditions in the calibration region compared to current conditions, we employed the kuenm_mop function from the kuenm package to calculate the mobility-oriented parity (MOP) metric. MOP analysis aids in identifying where strict extrapolation occurs, with values of 0 corresponding to such regions.

### 2.5 Assessment of the importance of environmental variables and their effect on climate change

The first step in conservation is to understand the relationship between the geographic distribution of taxa and environmental conditions. And this assessment can help conserve and restore endangered plants more scientifically and cost-effectively (Harapan et al., 2022). In this study, the contribution of environmental variables to the geographic distribution was determined by percent contribution and permutation importance. The percent contribution is the contribution of each environmental variable to the geographic distribution given by the MaxEnt model. And the permutation importance is the reduction of the AUC value obtained by randomly replacing the environmental variables in the training data. The larger reduction indicates that the model depends on the variable (Phillips, 2005). To understand how environmental variable affects the distributions, the MaxEnt model gives a response curve, showing the relationship between the probability of species presence and environmental variables. In this study, the environment suitable for the growth of *G. manshurica* was defined as the presence probability is larger than P (0.1899 in this study).

To elucidate the impact of future climate change on climate variables that affect the distribution of *G. manshurica*, the random selection of 1,000 points within the CPD was performed, followed by the generation of box plots for the relevant climate variables. In addition, for climate variables used in the modeling, we used a t-test to test whether the combination of SSP and the period is significantly different (p < 0.01) from the current climate. In this way, we can visualize what environmental stresses the CPD of *G. manshurica* will face under climate change.

### 2.6 Future range shift of elevation, latitude, and climate niche centroid

1,000 points were randomly selected in the FPD of *G. manshurica*. And the changes in elevation and latitude under climate change were calculated, and the ridge density map was drawn using the Ridgeline R package for representation.

The abundant center hypothesis (ACH) considers species most abundant close to the center of their geographic range. As the distance from the geographic centroid increases, the abundance is expected to decrease until reaching the distribution limit (Brown, 1984; Hengeveld, 1992). However, it has been demonstrated that ACH negatively correlates with environmental distance at the central condition of the climate niche centroid, rather than the geographic centroid of the species distribution (Martínez-Meyer et al., 2013). To reveal the influence of global warming on the *G. manshurica* climate niche centroid, we utilized the modeling climate factors used for PCA analytical modeling from sixteen climate scenario combinations. We retained the first 2 components, which explained cumulatively ≥ 95% of the total variance in the dataset. The climate niche centroid was derived from the mean of the retained PC layers as suitable by the thresholded MaxEnt result (Manthey et al., 2015).

### 2.7 Changes in landscape structure, landscape indexes, and landscape fragmentation

We extracted the land use data from the CPD. Then, we counted the area and proportion of different landscape types to analyze the changes in landscape structures in three years (1980, 2000, and 2020). Six indexes at the class level were calculated using Fragstats software. The landscape indexes were selected from three aspects. Patch area and number indexes: patch number (NP), patch density (PD), mean patch area (MPS); shape indexes: area-weighted mean of fractal dimension index (FRAC_AM); aggregation indexes: landscape division index (DIVISION) and aggregation index (AI) (Ran et al., 2019). The formulas of landscape indexes are described in the literature (Wu, 2007). The main landscape type suitable for *G. manshurica* under current climate conditions is grassland, so we chose this type to calculate the landscape fragmentation (LF). The moving window method was used, and the operation was referred to Ran et al. (2019). The result was classified with the equidistant method: extremely low fragmentation (0 ≤ LF < 0.2), low fragmentation (0.2 ≤ LF < 0.4), mid fragmentation (0.4 ≤ LF < 0.6), high fragmentation (0.6 ≤ LF < 0.8), and extremely high fragmentation (0.8 ≤ LF < 1). We used a raster calculator to map the spatial and temporal changes of landscape fragmentation in three years to identify increased and stabilized or reduced landscape fragmentation areas.

### 2.8 Analysis of habitat quality change

The InVEST 3.11.0-Habitat Quality model performed habitat quality in the study area. Referring to related studies (Chen et al., 2016; Yang et al., 2018b; Liang et al., 2020) and considering the actual situation of the study area and the research content, cultivated land, urban land, rural settlement, and other construction land were selected as threats. The following data were entered into the software: land use data in 1980, 2000, and 2020, threats factors layers, tables of threats factors (**Table S3**), and the table of sensitivity of land use types to threats (**Table S4**). The output results were the habitat quality in the study area in three years. After that, we extracted the habitat quality (HQ) results of three years based on the CPD of *G. manshurica* to obtain the habitat quality of this region. Then, we classified them into three levels: low-quality habitat (0 ≤ HQ < 0.33), medium-quality habitat (0.33 ≤ HQ < 0.66), and high-quality habitat (0.66 ≤ HQ < 1).

### 2.9 The determination of the PPAs and WTAs

ZONATION software proposed by Moilanen was applied to spatial priority conservation of species and large-scale spatial conservation planning. The principle is to generate hierarchical landscape priorities based on the raster’s biological values, considering the connectivity and priorities of biodiversity features (species, land use types, etc.) (Di Minin et al., 2014; Li et al., 2020). The combined application of MaxEnt and ZONATION has been extensively utilized in biodiversity conservation planning for both plants (Wang et al., 2015; Gouwakinnou et al., 2019) and animals (Yang et al., 2019). The input layers included the biotic factor layers, the landscape fragmentation layer, and the habitat quality layer in this study. The current and future results generated by the MaxEnt model constituted the biotic factor layers. The landscape fragmentation layer resulted from 2020 in Section 2.7 computed by Raster Calculator. The higher the value, the lower the degree of landscape fragmentation. And the habitat quality layer was the result of 2020 in Section 2.8. The cultivated and construction land in the 19 layers were removed because China is practicing the policy of preventing Non-Grain Production. We used the core-area zonation (CAZ) cell-removal rule to retain the core area of species distribution. And edge removal was used to promote the maintenance of structural habitat continuity in the removal process (Moilanen et al., 2009). The warp factor was set to 1 to remove one grid at a time for optimal running results. The weights of input layers were all set to 1. Other parameters were model default. Protecting 5% ~ 20% of the habitat has been shown to achieve more than 50% of species conservation (Margules and Pressey, 2000). According to the target, we set the top 20% of the result as the PPAs for practical purposes. Then, we graded the output results: the top 5% of the results were considered mandatory protection areas, 5% ~ 10% negotiated protection areas, and 10% ~ 20% partial protection areas (Yang et al., 2018a). We also collected data on national nature reserves in the study area to reveal the gap between PPAs and existing reserves.

China has vigorously advocated wild medicine materials tending in recent years to improve the quality of wild TCM and ensure the healthy development of the TCM industry. In the general rules, WTAs should be in the TCM primitive environment, with no pollution, good environmental quality, and business potential. Therefore, the remaining 80% of results were set as WTAs. County-level governments in China serve as crucial management units for conservation efforts (Yang et al., 2018a). The Getis-Ord Gi* was used to analyze the hotspots of the PPAs gap and WTAs for *G. manshurica* at the county level.

## 3 Result

### 3.1 The CPD of *G. manshurica*

In this study, we used the MaxEnt model to predict the potential distribution of *G. manshurica*. After kuenm package optimization, the best parameters were FC=QT and RM=2. The mean of training AUC and test AUC for the CPD and FPD under this parameter condition were greater than 0.9 (**Figure S2**), which can be used for the prediction.

**Figure 2:**
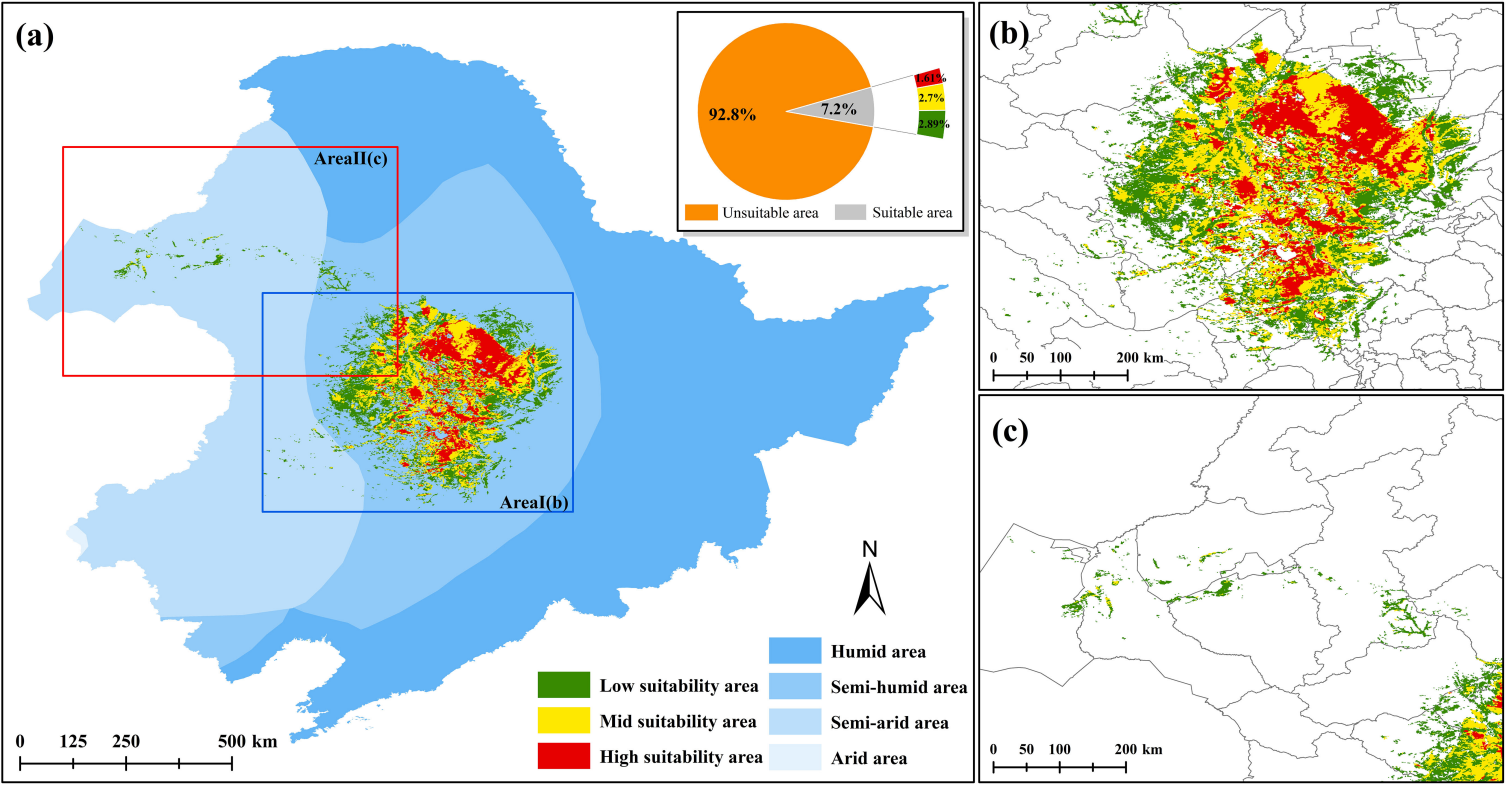
Current potential distribution of *G. manshurica* **(A)**. Current potential distribution of *G. manshurica;* **(B)**. Area I of current potential distribution of *G. manshurica*; **(C)**. Area II of current potential distribution of *G. manshurica*).

The CPD of *G. manshurica* is mainly distributed in the central part of the Songnen Plain (**Figure 2**), with an area of 8.83×10^4^ km^2^ (accounting for 7.19% of the study area). It is mainly distributed in the semi-humid area (97.64%), only a small part of the low and high suitability areas are distributed in the semi-arid area (2.36%). The high suitability area covers 1.97×10^4^ km^2^ (1.61%), mainly in Lindian, Anda, and Dorbod Mongol Autonomous County (Dorbod) of Daqing City. The mid suitability area is centered on the high suitability area and distributed radially outward, covering 3.31×10^4^ km^2^ (2.70%), mainly in Dorbod and Qiqihar. The low suitability area is centered on the mid suitability area and distributed sporadically outward, with an area of 3.54×10^4^ km^2^ (2.89%), mainly in Zalaite Banner, Nong’an, and Zhaodong.

### 3.2 Result of the changes in landscape structure, landscape indexes, and landscape fragmentation

**Figure 3:**
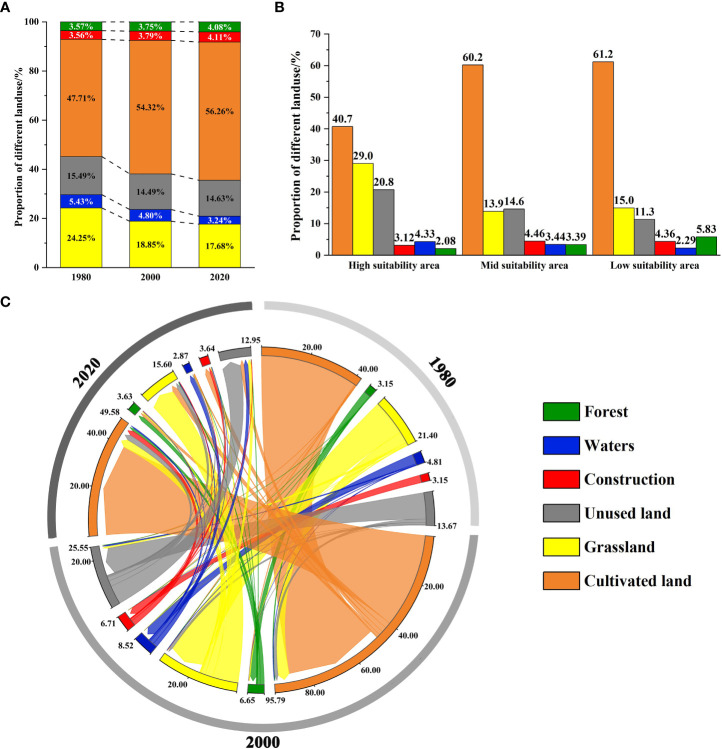
Landscape structure change map **(A)**. The proportion of landscape structure in the study area in 1980, 2000, and 2020; **(B)** The proportion of landscape structure in each grade of the CPD in 2020; **(C)** Landscape structure transformation matrix of the study area in 1980, 2000, and 2020.

We analyzed landscape structure changes in the study area from 1980 to 2020. Currently (2020), the main landscape type in the CPD is cultivated land (56.25%), which is much larger than the proportion of other landscape types. The proportion decreases as the level of the suitability area increases (**Figures 3A, B**). The trend of the change in the CPD in the last 40 years is that grassland and waters have decreased, while cultivated land, construction land, and forest land have increased, and unused land has no significant change. Compared with 1980, the grassland area decreased significantly by 22.25% in 2000 and 27.08% in 2020 (**Figure 3A**). Combined with the landscape structure shift matrix, it is clear that the intensified decline in grassland over the 40 years is mainly due to the significant expansion of cultivated land and the degradation of partly grassland to unused land (mainly marshland and saline land), followed by the conversion of forests, construction land and waters (**Figure 3C**; **Tables S5, S6**).

**Figure 4:**
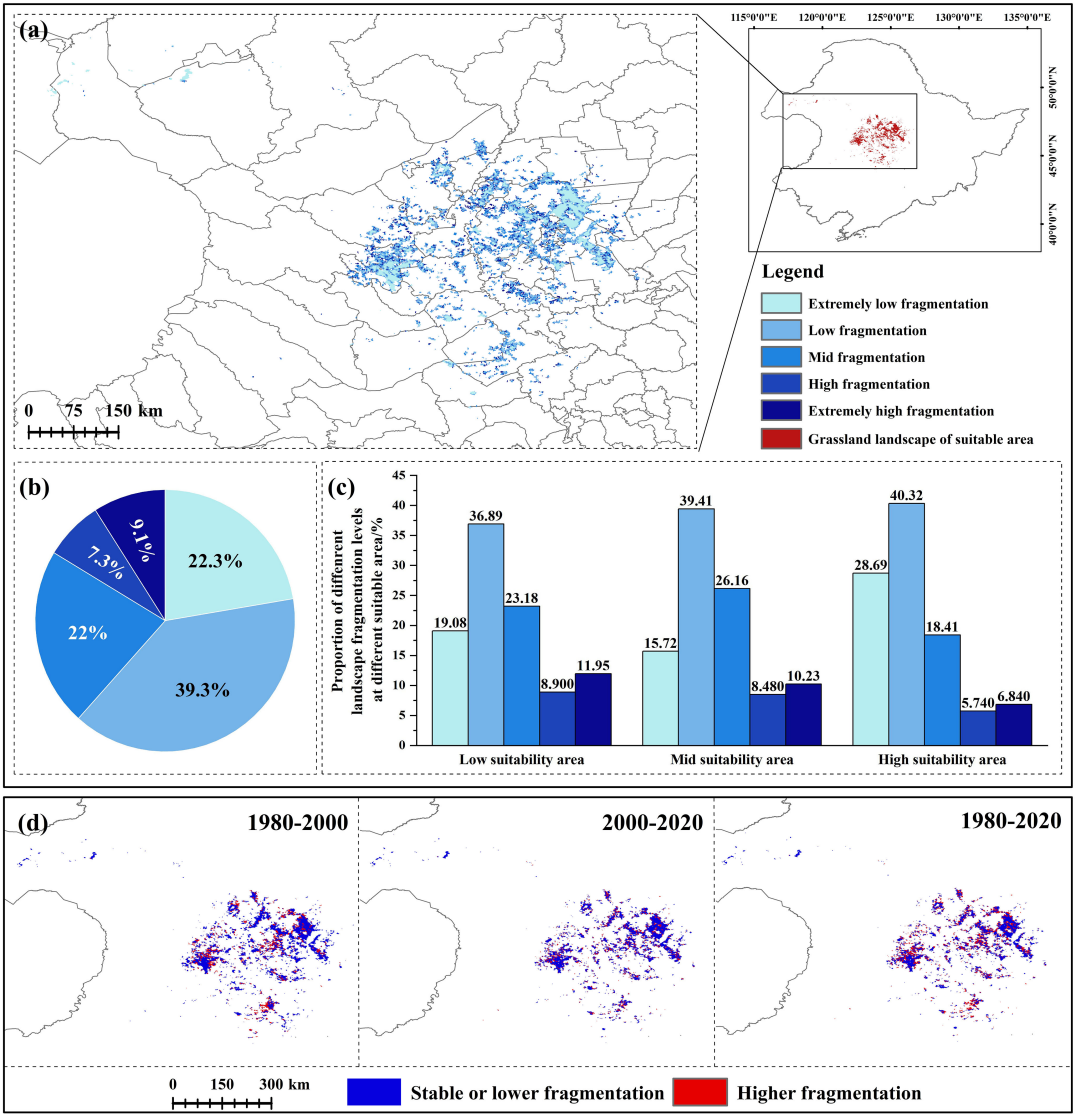
Landscape fragmentation of grassland in CPD **(A)**. landscape fragmentation in 2020; **(B)**. the proportion of different levels of landscape fragmentation; **(C)**. the proportion of different levels of landscape fragmentation in different levels of suitability area; **(D)**. landscape fragmentation changes in three periods.

Dynamic analysis of landscape indexes in the CPD showed that during 1980-2020, NP and PD decreased and then increased, while MPS decreased yearly, indicating that the patch area gradually decreased. Regarding the landscape shape index, FRAC_AM decreased yearly, indicating that humans have increasingly disturbed the grassland landscape. From the perspective of the aggregation indexes, DIVISION showed an increasing trend over the years, while AI showed an increasing trend, indicating that landscape aggregation decreased yearly and its integrity decreased (**Table S7**). In the past 40 years, the landscape fragmentation index increased and intensified everywhere. From 1980 to 2000, the area of each fragmentation level decreased due to the sharp decrease in grassland. The proportion of mid, high, and extremely high fragmentation increased, and the other decreased. From 2000 to 2020, the area of low and extremely high fragmentation increased, while the other decreased (**Table S8**). The spatial-temporal change analysis of the landscape fragmentation index of grassland types in CPD from 1980 to 2020 showed that the increased fragmentation mainly occurred in the high and mid suitability areas, mainly in Dorbod, Jalaid Banner, Lindian, and Horqin Right Front Banner (**Figure 4D**). Currently (2020), the area with extremely low fragmentation is generally in the center of the patch. Extremely high fragmentation is generally in the periphery. The area with extremely low and low fragmentation accounts for a relatively large proportion (61.62%) (**Figures 4A, C**). From the perspective of different levels of suitability area, with the increase of suitability, the proportion of the mid, high, and extremely high fragmentation showed a decreasing trend, while the other was contrary (**Figure 4B**). In summary, in the past 40 years, the grassland landscape in CPD decreased, and the fragmentation trend intensified. Although the area with low fragmentation in the high suitability area is relatively large at present, the intensified fragmentation mainly occurs in the high suitability area from the dynamic point of view, which is still a challenge for the population survival of the *G. manshurica*.

### 3.3 Results of habitat quality

**Figure 5:**
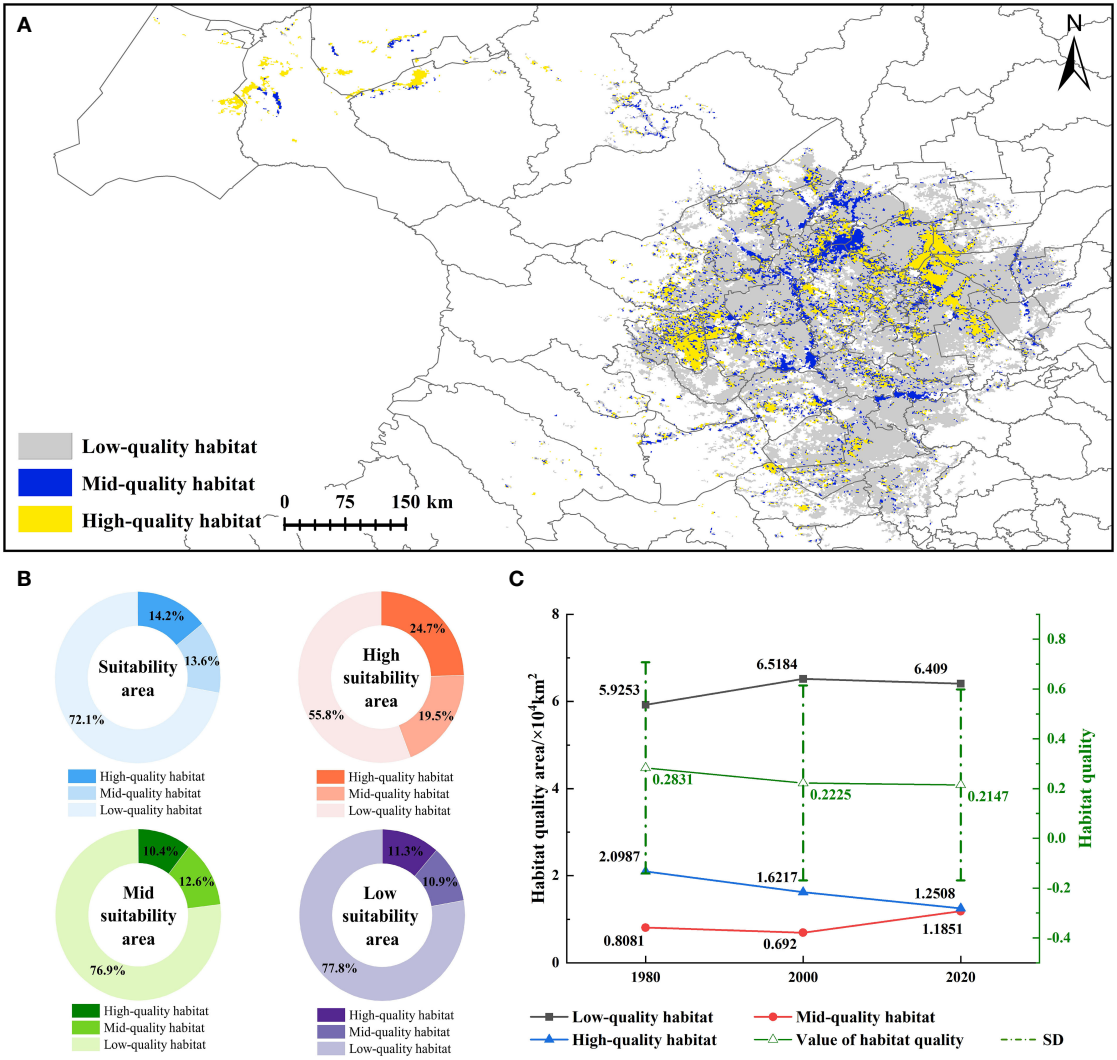
Habitat quality results **(A)**. habitat quality of CPD; **(B)**. statistical results of habitat quality at different levels; **(C)**. habitat quality indexes of CPD in 1980, 2000, and 2020.

In 1980 and 2000, the average habitat quality index in the CPD was 0.28 ± 0.42 and 0.22 ± 0.39, decreased by 21.41%. High-quality and mid-quality habitat areas decreased by 22.73% and 14.37%, while low-quality habitat increased by 10.01%. The average habitat quality index in 2020 was 0.21 ± 0.38, representing a 3.51% decrease compared to the index in 2000. The high-quality and low-quality habitats decreased by 22.87% and 0.18%. Conversely, mid-quality habitats increased significantly by 71.26% (**Figure 5C**). In 2020, high-quality and mid-quality habitats were 1.25×10^4^ km^2^ and 1.19×10^4^ km^2^, accounting for 14.14% and 13.40%, mainly distributed in Anda, Jalaid Qi, and Dorbod. Low-quality habitat accounted for 72.12%, mainly distributed in the Mongolian Autonomous County of Qian Gorlos and Dorbod. This result showed that low-quality habitat accounted for a large proportion, and the habitat quality of each level was embedded with each other, which was not conducive to the growth of species (**Figure 5A**). It can be seen that with the increase in habitat suitability, high-quality and mid-quality habitats gradually increased, while low-quality habitats gradually decreased (**Figure 5B**). In general, the habitat quality of CPD was very low, showing a declining trend. Moreover, the habitat quality decreased significantly from 1980 to 2000 compared with 2000 to 2020.

### 3.4 Dynamic change of spatial pattern of FPD

**Figure 6:**
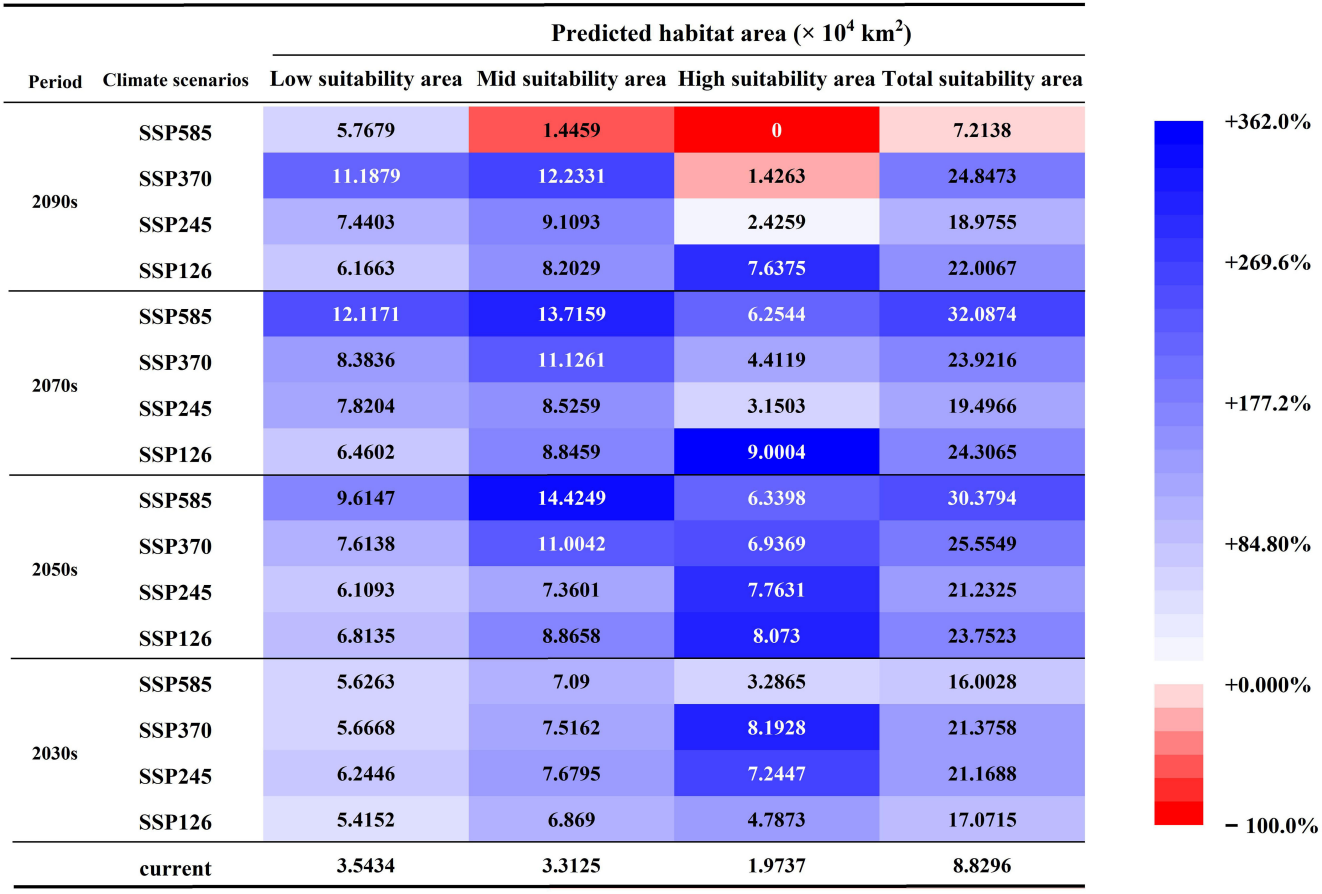
Suitability area of *G. manshurica* in Northeast China under different SSPs and periods. Blue in the cell represents an increase in the area of FPD compared to CPD. The bluer the color, the bigger the increase. Red represents a decrease in the area of FPDs compared to CPD. The more red the color, the larger the reduction.

**Figure 7:**
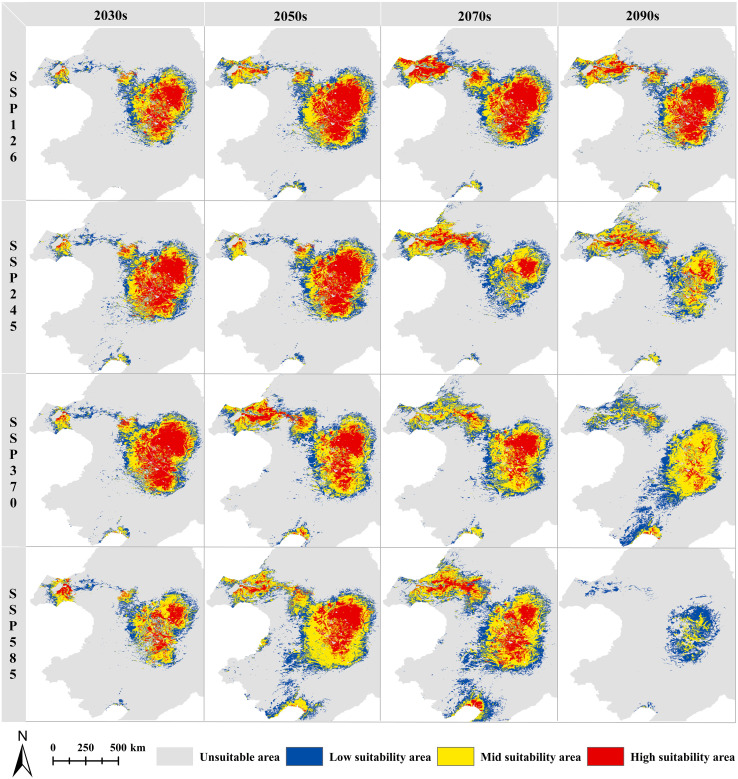
FPDs under different periods and SSPs for *G. manshurica*.

A comparative analysis of the CPD and FPDs is expected to show an overall increasing trend in the FPDs (**Figures 6**, **7**). For the low suitability areas, mid suitability areas, and total suitability areas, increasing trends are expected to be generally consistent under different SSPs in the 2030s, with growth rates between 52.82% and 76.23%. With the increase of SSPs, the area growth rate is expected to increase in the 2050s and 2070s. And in the 2090s, the growth rate is expected to increase first and then decrease. Note that in SSP585-2090s, the area is no longer increased. And the mid suitability area is expected to be 1.45×10^4^ km^2^, decreased by 56.35%. And the total suitability area is expected to be 7.21×10^4^ km^2^, decreased by 18.30%. For the high suitability area, with the increase of SSPs in the same year, the growth rate is expected to increase first and then decrease. At the same time, it is worth noting that in SSP370-2090s, the area is expected to stop growing and begin to decline, decreasing by 27.73%. During the SSP585-2090s, the high suitability area might completely disappear. In general, global warming is favorable to the growth of *G. manshurica* at the beginning. However, as the increase of years and SSPs, it poses an increasing threat to growth (**Figures 6**, **7**).

A more detailed map analysis showed that the central Songnen Plain is a stable potential distribution for *G. manshurica*. And the expansion will mainly be expected in the northwest and south of the CPD. With the increase of SSPs and years, the FPD in Liaohe Plain and Hulunbuir Plateau will be expected to increase year by year (except SSP585-2090s). The only shrinkage is expected in the western Songnen Plain (**Figure S3**). From the perspective of the division of dry and wet areas in China, generally speaking, compared with the CPD, the semi-arid area in the FPDs of different suitable levels will increase significantly, the humid area will increase slightly, and the area of the sub-humid area will decrease (**Figure S4**). The MOP analysis of the current climate conditions between the accessible region “M” and the projected area shows the strict extrapolation in the eastern, southern and central parts of the study area (**Figure S5**).

### 3.5 Environmental variables related to *G. manshurica*

**Figure 8:**
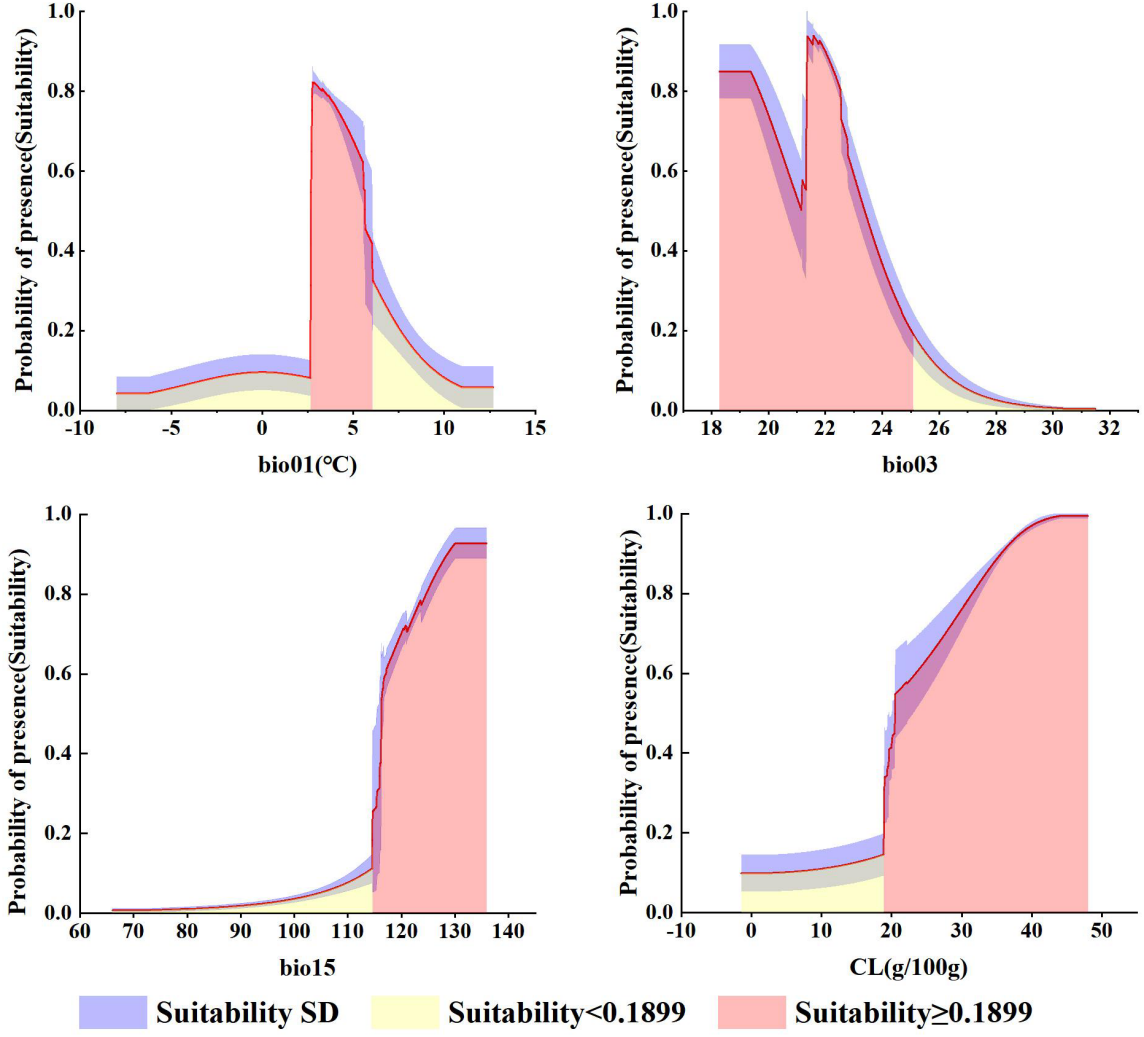
Response curves for environmental variables.

As can be seen from **Table S9**, bio15 (precipitation seasonality (CV)), bio03 (isothermality), bio01 (annual mean temperature), and CL (clay content) are the environmental variables affecting *G. manshurica*. The cumulative contribution percent of these environmental factors reached 88.2%, and the cumulative permutation importance reached 93.5%. Combined with the response curve given by the MaxEnt model, it can be known that there is a relationship between environmental variables and the presence probability of *G. manshurica*. Bio01 is -8 ~ 13°C, whereas the optimum temperature is 2.71 ~ 6.06°C (**Figure 8A**). The suitable range of bio03 is between 18.28 and 25.09. And there is a turning point at 21.16. From 18.28 to 21.16, habitat suitability decreased with increasing isothermality. While the value of isothermality from 21.16 to 25.09, habitat suitability suddenly increases, then gradually descends (**Figure 8B**). The optimum growth condition for *G. manshurica* in bio15 is between 114.63 and 130.05 (**Figure 8C**). The clay content of 18.88 ~ 48.02 g/100g is suitable for the growth of *G. manshurica* (**Figure 8D**).

**Figure 9:**
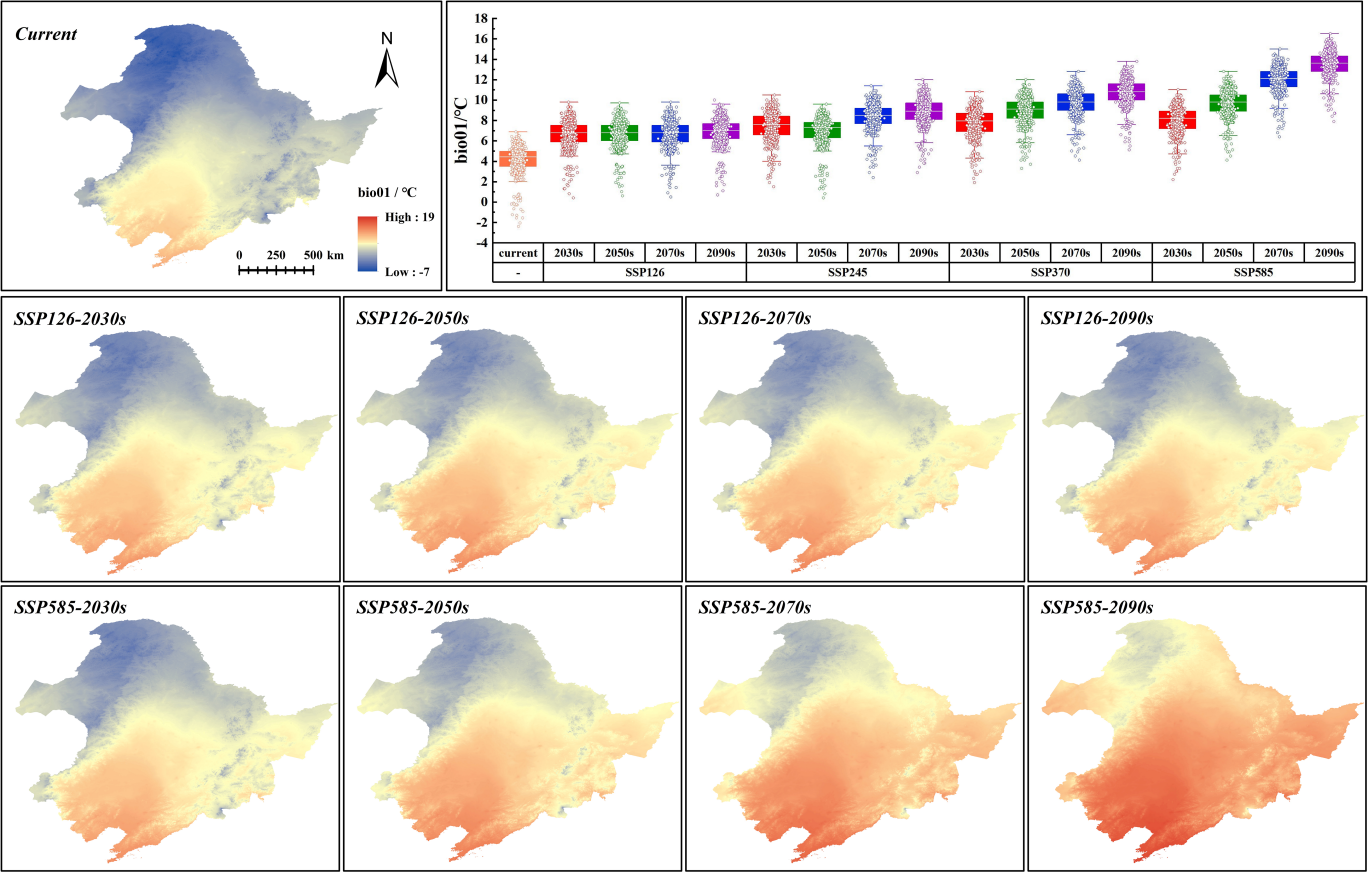
Bio01 value in SSP126 and SSP585 and box plots of bio01 change within CPD.

**Figure 10:**
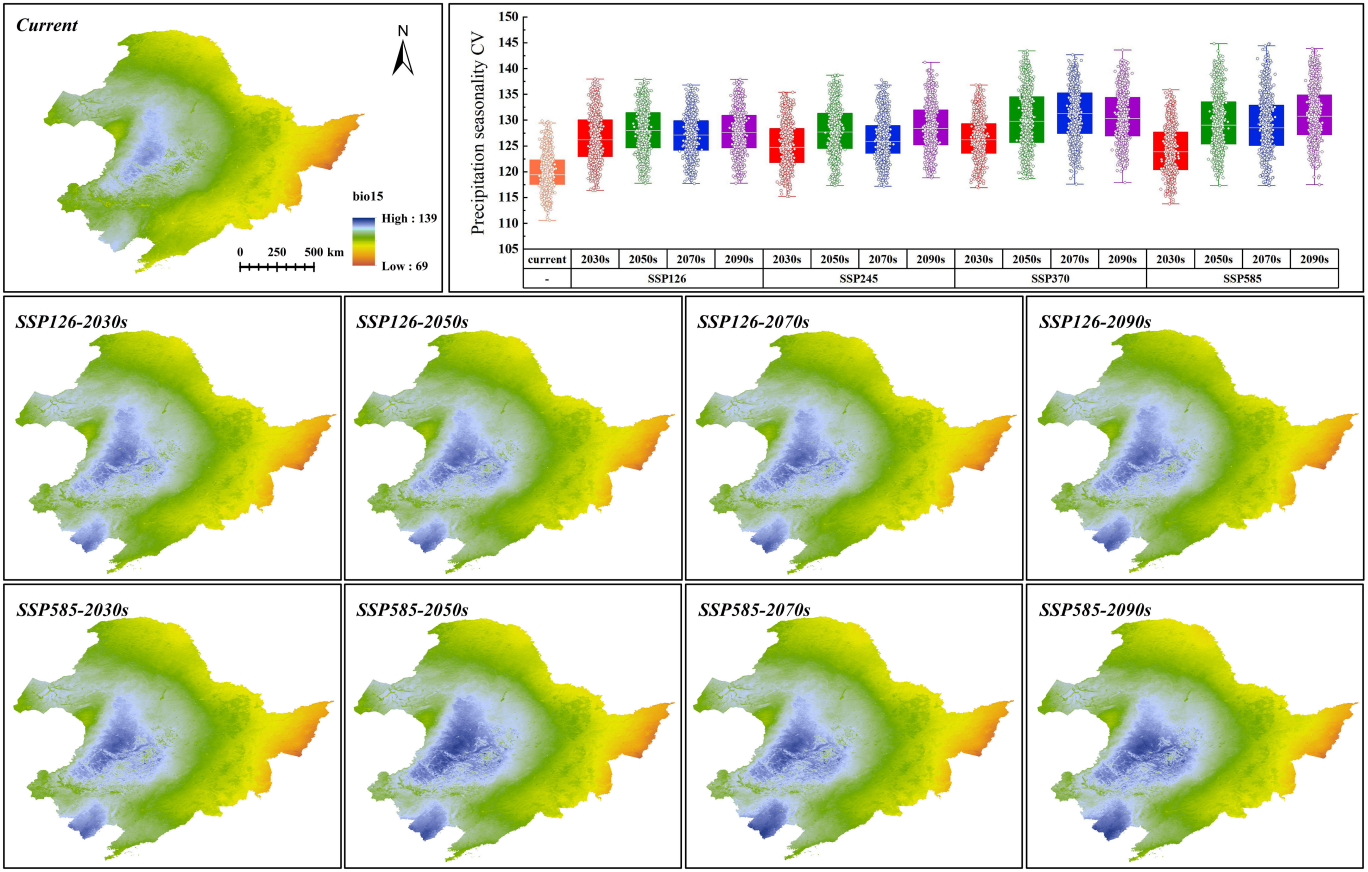
Bio15 value in SSP126 and SSP585 and box plots of bio15 change within CPD.

Three climate variables dominating the distribution of *G. manshurica* are affected differently by climate change. Bio01 and bio15 have extremely significant differences with the current climate conditions (p-value < 0.01), while bio03 is basically consistent (**Table S10**). With the increase of year and SSPs, the average bio01 in the CPD is expected to increase from 4.18°C to 13.51°C by the end of this century. Bio01, in the future, will gradually deviate from the suitable range in the CPD based on the response curve of environmental factors (**Figures 8**, **9**). Although bio15 under future climate conditions significantly differs from current climate conditions, its average value is still in the appropriate range (**Figures 8**, **10**). In conclusion, with climate change, the future pressure on the CPD of *G. manshurica* is due to the growth of bio01.

Bio01 in the study area decreases with increasing latitude. Climate change significantly affects it and has obvious changes with increasing SSPs. At the end of this century of the SSP126 and SSP585, the mean values increase to 6.59°C and 12.29°C (**Figure 9**), which are both higher than the tolerance of *G. manshurica* for bio01. Bio15 shows a downward trend with the Songnen Plain as the center. And it also shows a trend affected by climate warming. At the end of this century of the SSP126 and SSP585, the mean value increases to 114.82 and 115.29 (**Figure 10**), which is still within the suitable range. Therefore, we believe that the expansion of the suitable area is also due to increasing bio01.

### 3.6 Future range shift of elevation, latitude, and climate niche centroids

In the future, the average elevation of the FPD of *G. manshurica* will increase. The current average elevation is 176.99 m. Under low carbon emission scenarios (SSP126 and SSP245), the average elevation is expected to increase yearly. In SSP245-2090s, the average elevation is expected to change the most, rising to 415.68 m. However, under the high carbon emission scenario (SSP370 and SSP585), the average elevation is expected to increase in the first three periods and decrease in the 2090s. There is no apparent change in the mean latitude. Under the same SSPs, the mean shows an increasing and then decreasing trend. The mean latitude is lower than the current in the SSP370-2090s, SSP585-2050s, and SSP585-2090s (**Figure S6**).

**Figure 11:**
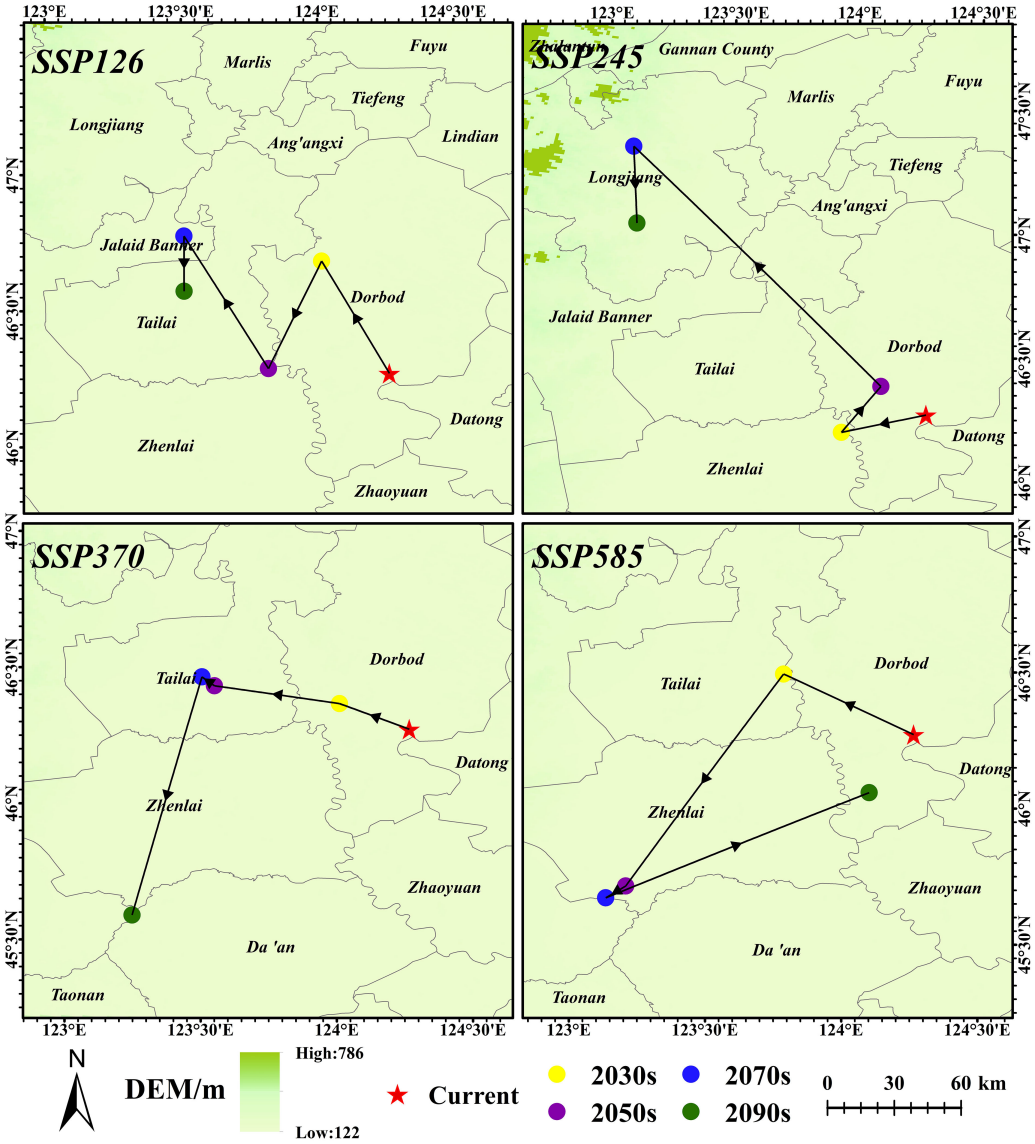
Climatic niche centroid of *G. manshurica* in FPDs.

Currently, the climate niche centroid is 124.27° E, 46.27° N (in Dorbod County). Climate niche centroid migrates to the northwest under the low carbon emission scenarios (SSP126 and SSP245). In the SSP245-2070s, the climate niche centroid is expected to shift the farthest to the northwest (150.67 km) to 123.09° E, 47.36° N (in Longjiang County). In SSP370, the migration trend is first to the northwest and then to the southwest over time (**Figure 11**). Under extreme climatic conditions (SSP585), with the increase of years, the climate niche centroid is expected to migrate to the northwest, then to the southwest, and finally return to Dorbod County (124.10° E, 46.06° N).

### 3.7 Planing the PPAs and WTAs

**Figure 12:**
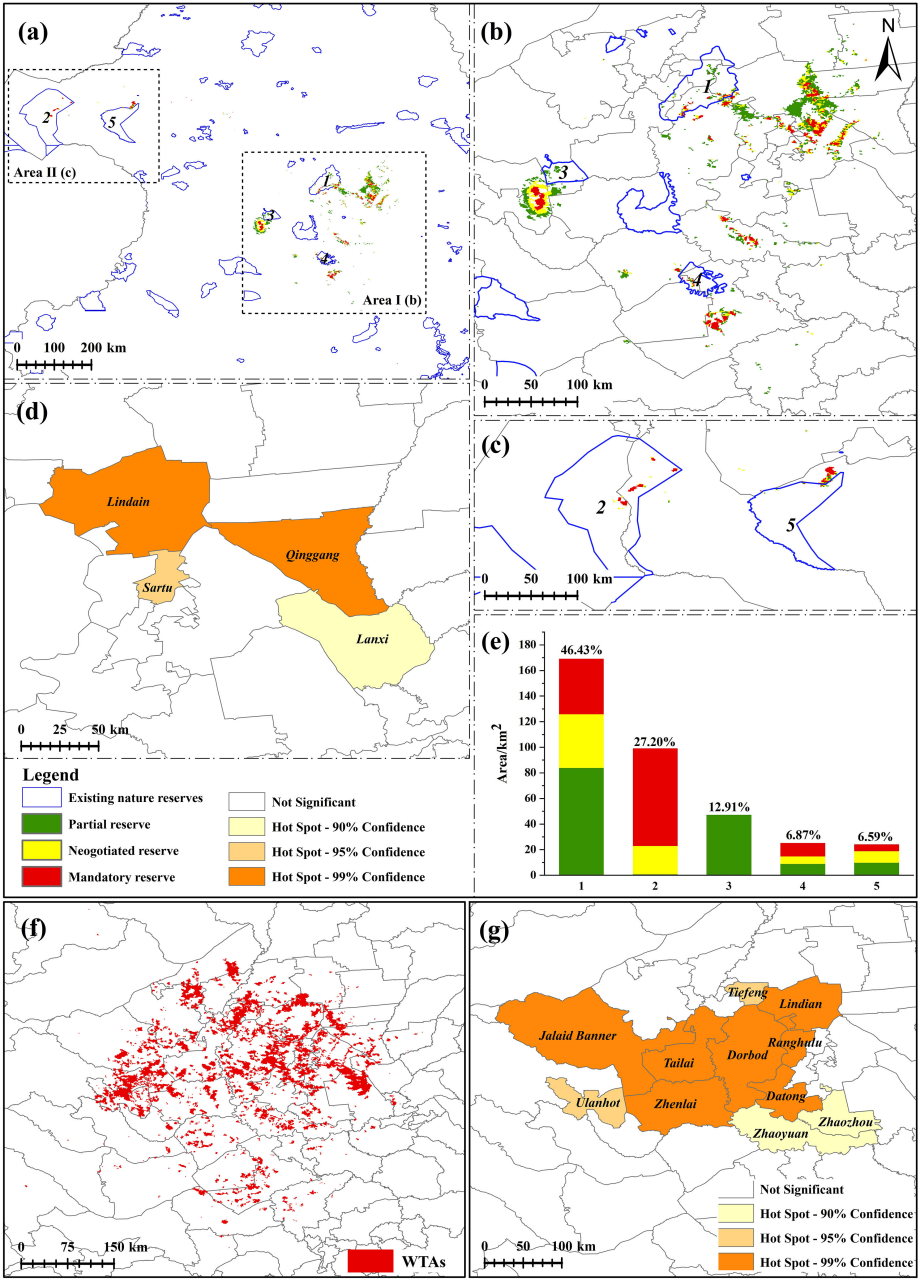
PPAs and WTAs of *G. manshurica* **(A)**. PPAs compared with existing nature reserves; **(B)**. PPAs compared with existing nature reserves of Area I; **(C)**. PPAs compared with existing nature reserves of Area II; **(D)**. hotspots of PPAs; **(E)**. contribution of existing nature reserves to different levels of PPAs; **(F)**. WTAs of *G manshurica*; **(G)**. hotspots of the WTAs). 1: Heilongjiang Zhalong National Nature Reserve; 2: Inner Mongolia Hulun Lake National Nature Reserve; 3: Inner Mongolia Tumuji National Nature Reserve; 4: Jilin Chagan Lake National Nature Reserve; 5: Inner Mongolia Huihe National Nature Reserve.

According to the results of the ZONATION, the PPAs under climate change are 3,823 km^2^ (**Figure 12A**). Only 364 km^2^ of PPAs are protected, accounting for 9.52% of PPAs, and only 0.41% of CPD. It is mainly distributed in Songnen Plain (Area I) and Hulunbuir Plateau (Area II) (**Figures 12B, C**). Mandatory and negotiated protection areas are 956 km^2^, and partial protection areas are 1,911 km^2^. Five protected areas contribute to the conservation of *G. manshurica*, and Heilongjiang Zhalong National Nature Reserve has the highest contribution, accounting for 46.43%. Inner Mongolia Hulun Lake National Nature Reserve followed, accounting for 27.20% (**Figure 12E**). Simultaneously, these two reserves provide the largest mandatory and negotiated protection area, accounting for 88.82% and 81.01% of the total area of the mandatory and negotiated protection area. We used Getis-Ord Gi* hotspot analysis for PPAs gaps to delimit county-level protection. The results showed 4 hotspots in PPAs, including two core hotspots (Lindian and Qinggang County), one sub-hotspot (Sartu), and one marginal hotspot (Lanxi) (**Figure 12D**). The area of WTAs is 15,291 km^2^, and there are 11 hot spots in WTAs. Seven core hotspots (Lindian, Datong, Jalaid Banner, Ranghulu, Zhenlai, Tailai, and Dorbod), two sub-hotspots (Ulanhot and Tiefeng), and two marginal hotspots (Zhaoyuan and Zhaozhou) are planned to as the site for the wild tending of *G. manshurica* (**Figures 12F, G**).

## 4 Discussion

### 4.1 Effects of environmental variables on the distribution of suitable areas of *G. manshurica*

The first step in conservation is to understand the relationship between the geographical distribution of taxa and environmental conditions (Harapan et al., 2022). The results show that precipitation (bio15) and temperature (bio01 and bio03) are the main factors limiting the distribution of *G. manshurica*. Under the background of climate change, precipitation, and temperature changes will play an important role in the dynamic evolution of plant communities.

The change in precipitation pattern significantly affects plant growth and regeneration. Growing evidence suggests that precipitation variability and extremes exert a more significant influence on ecosystem processes than the average precipitation level (Zeppel et al., 2014). Bio15 is a measure of the variation in monthly precipitation totals over the course of the year, which affect phenology, such as fruit, leaf, and early/late wood development. Thus, bio15 is important for species growth (Misson et al., 2011). The results show that bio15 positively affects the growth of *G. manshurica*. The seasonal rainfall in Northeast China varies greatly, mainly in summer and fall and least in winter (Yao et al., 2017). As a narrow species in Northeast China, bio15 in the suitable area is higher than in the unsuitable area, indicating that precipitation distribution in a year in the suitable area is more uneven, and the precipitation in summer and fall would be more concentrated. This result is consistent with our field survey results and literature records that *G. manshurica* is mainly concentrated in seasonally waterlogged areas of local higher elevation (Liu et al., 1995).

Clay content is also a key factor for *G. manshurica*, which works by coupling with precipitation. The change in precipitation pattern has an important effect on soil water. The size and distribution of precipitation events further interact with local topography and soil, affecting the extent and depth of soil water replenishment (Reynolds et al., 2004). Soils with high clay content generally have a great water-holding capacity and increase the utilization efficiency of precipitation, which positively affects water absorption, plant water status, evaporation, cooling, and carbon gain (Ismail and Ozawa, 2007; Maharjan et al., 2022). Seed germination of *G. manshurica* requires a high-humidity environment. In the early stage of individual development, *G. manshurica* seeds have some characteristics of original aquatic macrophytes, such as radicle hysteresis and hypocotyl hair at the root tip. Therefore, long-term moist conditions are required. At the same time, within one month after germination, the seedlings will be easily exposed to dry soil and die due to the lack of water in the shallow soil layer (Meng et al., 2011). Therefore, soil with high clay content is conducive to seed germination and promotes the establishment and continuation of population.

Temperature is a significant factor in controlling plant growth and development, influencing plant metabolism, regulating nutrient uptake, and determining the biogeographic distribution of organisms. Bio03 is the diurnal temperature range vs. the annual temperature range, which reflects the time and amplitude of temperature change. And it is related to the length of the growing season and affects the distribution of species (O’donnell and Ignizio, 2012; Rawat et al., 2022). The suitable range of *G. manshurica* is 18.28 ~ 25.09 (less than 100), indicating that the annual temperature range is more drastic than the diurnal temperature range in the suitable area. Bio01 is one of the most important environmental variables affecting above-ground biomass and species richness. The earliest seed at the top of the stem of *G. manshurica* is usually fully ripe in late September. Then the above-ground part of the plant grows until it dies from frost. In Northeast China, the first frost date in Songnen Plain is in late September, which is later than that in the Greater and Lesser Khingan Range, Changbai Mountains, and other Northeast China. The late first frost date is beneficial to the seed fruiting of *G. manshurica*. A good deal of high-maturity seeds obtained can ensure the natural regeneration of the population. Therefore, the four dominant environmental variables can explain why *G. manshurica* only distributes in the narrow area of Songnen Plain in Northeast China.

### 4.2 Distribution pattern and change of suitable areas of *G. manshurica*

*G. manshurica* has very strict requirements for its growth environment (climate and soil), with narrow environmental tolerance. And it is a typical grassland plant. It often occurs in local highlands in seasonally flooded areas (Liu, 1988). At the same time, it requires a high temperature and high humidity environment during seed germination. The seed germination and seedling growth process is extremely slow, and such environmental conditions must be maintained for one month afterward (Meng et al., 2011). Few areas in the Songnen Plain can simultaneously meet such habitats (Liu, 1988). The results of this study show that soil factors (such as clay content and pH) are also environmental factors limiting the distribution of *G. manshurica*. The Songnen Plain is one of the world’s three major saline-alkali lands, and the soil is severely saline-alkali and desertified (Yang and Zhang, 2010). The salinization of soil will reduce the suitability value of *G. manshurica* in the region. At the same time, land use changes will also affect the climate (Yin, 2023). And our research results also show that a large amount of cultivated land has replaced grassland areas. These results make it even more difficult to meet the optimal climate conditions (high temperature and high humidity) for the growth of *G. manshurica*. Thus, it is plausible that the limited spatial extent of optimal environmental conditions for *G. manshurica* growth in Northeast China may explain the small proportion of high suitability areas.

*G. manshurica* is confined to the Songnen Plain in Northeast China, an area vulnerable to the impacts of climate change characterized by the high frequency and intensity of extreme weather events (Ji et al., 2006). Recent studies indicate that the region has experienced a significant increase in average temperature and a corresponding decrease in annual precipitation over the last 50 years, posing a significant threat to the area’s biodiversity (Wang et al., 2022b). Our study found that global warming is initially expected to favor *G. manshurica*, but as years and carbon emissions increase, it begins to threaten growth. Our t-test seems to provide a plausible explanation for this trend. Bio01 and bio15, affecting the distribution of *G. manshurica*, have significant differences between the future and the current, while bio03 has no significant difference. Bio15 values have been in the suitable range in all climate scenarios. While bio01 is only at the tolerance threshold in the SSP126, it adversely affected *G. manshurica* in the other three climate scenarios. Therefore, it is speculated that bio01 and bio15 may determine the future distribution of *G. manshurica*. Specifically, the combination of bio15 and bio01 is conducive to *G. manshurica* under SSP126, SSP245, and SSP370. Even though bio01 is unfavorable to growth, its minor negative effects may be covered by bio15 with more positive effects. As a result, under these three climate scenarios, the suitable area is expected to show an increasing trend, expanding from Songnen Plain to Liaohe Plain and Hulunbuir Plateau. However, in the SSP585, the value of bio01 will increase abnormally, which greatly exceeds the thermal tolerance of *G. manshurica*. The negative effects will gradually exceed the positive effects, resulting in the growth rate of the suitable area decreasing year by year, and the high suitability area will be expected to disappear completely in the SSP585-2090s.

### 4.3 Future range shift of elevation, latitude, and climate niche centroid

The interplay of adaptation and migration has played a central role in biological responses to climate change (Davis and Shaw, 2001). Most species are projected to shift towards higher latitudes and elevations in response to climatic shifts. However, this response can vary significantly within taxonomic groups (Chen et al., 2011). According to a global literature survey, until the early 21st century, nearly 20% of species altered their ranges towards lower elevations and/or southern latitudes in response to rising temperatures (Parmesan and Yohe, 2003; Lenoir et al., 2010).

For *G. manshurica*, the elevation and climate niche centroid are projected to respond to warming, while latitude trends are less significant. Under various climate scenarios, there is a trend for *G. manshurica* to move to higher elevations, albeit with a weak elevation increase trend in the later stages of high carbon emission scenarios. Moreover, the migration trend for the climatic niche centroid indicates inconsistency under different climate scenarios. Under low carbon emission scenarios, the centroid migrates northwestward with increased years. In contrast, under high carbon emission scenarios, the centroid migrates exceptionally, shifting first to the northwest, then moving southwestward before finally returning to the original administrative region of the current centroid position. This phenomenon may be due to a decrease in the total suitable habitat area of *G. manshurica* under extreme climate scenarios. Thus, the remaining populations may face significant threats to their reproduction and survival and may only be distributed within CPD. Northeast China is greatly affected by climate warming (Zhou, 2015). The reason for the shifting of *G. manshurica*’s climate ecological niche centroid may be that as temperatures rise, potential evapotranspiration will increase, water consumption will become more significant, and the phenomenon of warm-drying will become more apparent (Lian et al, 2001). Under future climate change scenarios, the current semi-humid areas in Northeast China are likely to become semi-arid regions (Ma et al., 2019). The shift of climatic niche centroid reflects core habitat shifts. That implies increasing protection of the CPD is necessary and critical for the conservation of *G. manshurica* and the ecosystem structure. While it is unlikely that a few dominant environmental variables and their tolerance thresholds will explain the aspects of species distribution, some experience supports a strong influence of these variables in determining geographic distribution (Hoffmann et al., 2005; Markle and Kozak, 2018; Tagliari et al., 2021).

It is worth noting that the climatic niche centroid we used differs from the geographical centroid in previous studies. By comparing the migration results of the two centroids, we can see that the migration trend is basically the same, but the migration of the geographical centroid is larger and farther away (**Figure S7**). Specifically, the geographical centroid only represents the shape center of the suitable area, which has no ecological significance. The climate niche centroid is the weighted average center of key climatic variables that affect the distribution of *G. manshurica* in the suitable areas, representing the highest region in the climate-suitable area. Moreover, the population in the climate niche centroid has the largest genetic variation, which is conducive to improving the adaptation potential to climate change (Hoffmann et al., 2005; Leimu et al., 2010; Lira‐Noriega and Manthey, 2014). Therefore, it is more reasonable to use climate niche centroid to study the migration trend of *G. manshurica* under climate change.

### 4.4 Effects of landscape pattern and habitat changes on the distribution of *G. manshurica*

Climate change is not the only factor threatening the survival of *G. manshurica*. The disturbance from humans is also increasing. Previous studies showed that *G. manshurica*, originally distributed throughout Northeast Plain, was only maintained for half a century. In the 1990s, only a few individuals were available in the Songnen Plain of Heilongjiang province, and nowhere else can be found (Liu, 1988). In the past 40 years, the landscape structure of CPD changed. Cultivated landscapes replaced many grassland landscapes, and the habitat was degraded. Moreover, the habitat quality was extremely low and developed in a lower direction. These results will lead directly to a decrease in suitable habitats. Another reason for the decline in grassland landscapes is degrading into marshland and saline-alkali land. Songnen Plain is one of the three saline-alkali regions in the world. In addition to natural factors, human factors such as unreasonable wasteland reclamation, grazing, and mowing will also cause secondary salinization, accelerating the alkalinization process again (Wang et al., 2009). *G. manshurica* can’t survive on salinized land. Thus, the increase in salinized land will further reduce its potential habitat. Therefore, the loss of habitat caused by human disturbance has an extremely adverse effect on the reproduction of the population, which would further aggravate the decline of the species.

Human disturbance has also increased habitat fragmentation and this ecological process is increasingly becoming a major threat to biodiversity (Fahrig, 2003; Fischer and Lindenmayer, 2007; Wu and Lv, 2008). From 1980 to 2020, CPD was affected by habitat fragmentation. Habitat fragmentation in synergy with climate change produces more severe negative effects, reducing the ability of species to track rapid climate change (Leimu et al., 2010; Meng et al., 2011). There are two reasons to interpret this phenomenon. First, the range shift to habitats with optimal climate conditions is impeded in a fragmented landscape. Second, the reduction of genetic variation in fragmented populations is predicted to reduce the adaptive potential of species under climate change (Leimu et al., 2010). For plants, migration occurs in the form of propagules and pollens, which requires habitat patches to be tightly connected enough to allow genes to flow between populations (Davis and Shaw, 2001).

The seeds of *G. manshurica* are small and light, belonging to the short-distance seed. Under natural conditions, the longevity of seeds is up to two years, which is short-lived (Liu et al., 1995). It isn’t easy to realize natural population regeneration by soil seed banks, which can only rely on the seed rain of plants. Moreover, its seeds mature in late September at the earliest. However, mowing begins mid to late August, making it impossible to form mature seeds due to the developed animal husbandry in the Songnen Plain (Sun et al., 2003). The natural regeneration of the population will be limited to some extent because the amount of seed rain is negatively affected by habitat fragmentation and the direct human factor, thus affecting the propagation, spread, and continuation of species (Li et al., 2007; Jesus et al., 2012; Liao et al., 2013).

Regarding genetic variation, habitat fragmentation increases the chance of random genetic drift and inbreeding rates and reduces inter-specific gene flow (Yu et al., 2019). The reproductive structure of *G. manshurica* shapes its self-pollination mode (Sun et al., 2003). Species predominantly self-interbreeding tend to have low levels of intraspecific genetic variation, affecting the gene flow between individuals and populations (Durka et al., 2013). In addition, as a narrow-range species, its genetic diversity at the population level may be significantly lower than its widespread counterparts. It may be more sensitive to the loss of variation due to genetic bottlenecks, resulting in its poor ability to adapt to new environments (Lavergne et al., 2004; Willis and MacDonald, 2011). Thus, it may not cope with future drastic climate changes. In particular, *G. manshurica* has the characteristics of rarity and specialization, which may increase the risk of species extinction. At worst, these two characteristics have a synergistic effect leading to more likely than common species to disappear from the regional species pool (Davies et al., 2004; Platts et al., 2014).

When plants face new selective pressures, such as climate change, there are three ways to respond: death, migration, and adaption (Leimu et al., 2010). Genetic limitations to adaptation combined with land use changes, which will hinder gene flow, may reduce the rate of adaptation significantly below the rate required for climate change (Davis and Shaw, 2001; Opdam and Wascher, 2004). Furthermore, the population tolerance and resilience of *G. manshurica* may be reduced under climate change. Under the double pressure of climate change and human disturbance, its migration may become more difficult. These factors may work alone or together, leading to an extinction vortex and the extinction of the population ultimately. Therefore, measuring the relationship between product development and wild conservation is necessary.

### 4.5 Identification of PPAs and WTAs under climate change

Although FPD is expected to increase, part of CPD may fall outside the climatic niche under the high carbon emission scenario. At the same time, land use change, low habitat quality, and poor-connectivity landscape will pose significant obstacles to the migration of the population. If facing the pressures from prevented migration, the inability to adapt to climate, and constant and destructive digging, the population of *G. manshurica* will suffer a great calamity. Therefore, our study provides a theoretical framework for conserving the wild resources of *G. manshurica*. If reserves are managed well, they could be an efficient and effective means to address biodiversity loss, which can buffer communities from the effects of climate change (He and Cliquet, 2020). The existing reserves provide only 9.52% of PPAs. Heilongjiang Zhalong National Nature Reserve and Inner Mongolia Hulun Lake National Nature Reserve, a stable climate refuge under climate change, contribute a large area of protection to the PPAs of *G. manshurica*, which can provide a good environment for population reproduction. But there are still gaps in the PPAs. Due to the difficulty of establishing a large reserve, further efforts are needed to extend the existing reserves (Wang et al., 2022a). In China, local governments are the main participants and law enforcers in biological protection. We analyzed biological protection hotspots at the county level to fill the protection gap. The results showed that Lindian, Qinggang, Sartu, and Lanxi are the hotspots of the PPAs. These cities with high-quality habitats and high-connected landscapes are suitable for *G. manshurica* and still provide a stable refuge in climate change. Therefore, the strict conservation of the wild population and natural habitat of *G. manshurica* in these cities should be needed to achieve sustainable development of the species.

On the other hand, as one of the original species of Gentianae Radix et Rhizoma, *G. manshurica* is of the best quality. However, with the increasing use of medicine, the limited wild resources are in danger. The domestication of *G. manshurica* has not been successful (Wang, 2005). The most suitable cultivation method for the species is to carry out wild tending in its native natural ecological communities because of the high susceptibility to pests and diseases and low survival rate planted out of its native habitat. The hotspot analysis showed 11 counties where wild cultivation could be carried out, including Lindian, Datong, Jalaid Banner, etc. Under the conditions of the natural ecological environment, wild tending can balance the contradiction between short supply and increasing demand of the TCM, maintain the expansion and protection of populations, solve the contradiction between medicinal plant production and ecological diversity conservation, maximize the best combination of yield and quality, and promote the sustainable utilization of the resources.

There is no doubt that the establishment of PPAs and WTAs is not enough to conserve *G. manshurica*. In addition, we propose the following recommendations. First, to dynamically monitor changes in the distribution, it is necessary to conduct regular surveys and threat assessments of its resources. Also, conducting this work will improve the prediction result by reducing sampling bias since model accuracy improves with increasing sample size, making conservation planning and other applications more scientific and rational (Hernandez et al., 2006). Second, based on the results of this study, germplasm collection should be conducted within the high suitability area. Given that germplasm resources cannot be recreated once they disappear, retaining as much genetic diversity as possible to mitigate the impact of climate change. Last, strengthen the research on the biology, cultivation, domestication, and wild tending of *G. manshurica*. In particular, the significant effects of climate change should be considered when carrying out related work. In these ways, we provide a reference for formulating related policies and taking countermeasures for adapting to climate change and human disturbances, ultimately achieving the conservation of this species.

## 5 Conclusion

In this study, we used the optimized MaxEnt model to successfully predict the current and future suitable areas of *Gentiana manshurica*. And we analyzed the habitat quality and landscape fragmentation based on land use data. Finally, the above results were used to construct a protective planning framework. This work is time-consuming and complicated due to the endangered state of the wild resource and the difficulty of identifying. Moreover, improved distribution of information can facilitate and promote the sustainable utilization of *G. manshurica* resources, which can lead to salvation in the knowledge gap, and budgetary costs associated with field surveys. Additionally, accurate information can enable evidence-based initiatives to strategize against environmental disturbance and climate change. The results show that although the potential suitability area of *G. manshurica* is relatively optimistic in the low carbon emission scenario, the worse results obtained in the SSP585 may be more reasonable and realistic because this scenario is most consistent with the recent trends in China. At the same time, human activities impact on the species and habitat cannot be ignored. Thus, our research is a reminder of the urgent need to reduce global carbon emissions to reduce the negative effects of climate change. In addition, human beings should also carry out protective activities and make multi-pronged efforts to maximize the stability and reproduction of the population. Finally, our results support the suggestions of relevant scholars, believing that it is necessary to upgrade *G. manshurica* to the second-level national key-protected medicinal material in China.

## Data availability statement

The original contributions presented in the study are included in the article/**Supplementary Material**. Further inquiries can be directed to the corresponding authors.

## Author contributions

HZ, BC, BZ, XYZho, XYZha, XXZ, and JW participated in the study design and analysis of the manuscript. HZ wrote the manuscript. BC, BZ, XYZho, and XYZha revised and processed the manuscript. XZ and JW gave valuable comments in writing the manuscript, and supervision and financial support. All authors contributed to the article and approved the submitted version.
