# Supplementary material for: Conservation planning for the endemic and endangered medicinal plants under the climate change and human disturbance: a case study of Gentiana manshurica in China
Source: Front Plant Sci. 2023 Jul 26;14:1184556. doi: 10.3389/fpls.2023.1184556 (PMC10410459; doi:10.3389/fpls.2023.1184556)
Supplement: Supplementary file 1 [file DataSheet_1.pdf]

Supplementary Material

Conservation planning for the endemic and endangered medicinal plants under the climate change and human disturbance: a case study of *Gentiana manshurica* in China

Hui Zou[1], Bingrui Chen[1], Boyan Zhang[1], Xinyu Zhou[1], Xiyuan Zhang[1], Xinxin Zhang[1*], Jianwei Wang[2*]

*** Correspondence:** Xinxin Zhang: hsdzxx2021@163.com; Jianwei Wang: wangjianweilikai@163.com

Supplementary Table 1. Environmental variables used in MaxEnt model.

Category	Variable	Description	Unit
Climate	bio01	Annual mean temperature	℃
	bio02	Annual mean diurnal range	℃
	bio03	Isothermality	%
	bio04	Temperature seasonality	℃
	bio05	Max temperature of warmest month	℃
	bio06	Min temperature of coldest month	℃
	bio07	Annual temperature range	℃
	bio08	Mean temperature of wettest quarter	℃
	bio09	Mean temperature of driest quarter	℃
	bio10	Mean temperature of warmest quarter	℃
	bio11	Mean temperature of coldest quarter	℃
	bio12	Annual precipitation	mm
	bio13	Precipitation of wettest month	mm
	bio14	Precipitation of driest month	mm
	bio15	Precipitation seasonality (CV)	-
	bio16	Precipitation of wettest quarter	mm
	bio17	Precipitation of driest quarter	mm
	bio18	Precipitation of warmest quarter	mm

	bio19	Precipitation of coldest quarter	mm
	PH	pH Value (H_2O)	-
	SOM	Soil Organic Matter	g/100 g
	TN	Total N	g/100 g
	TP	Total P	g/100 g
	TK	Total K	g/100 g
Soil	CEC	Cation Exchange Capacity (CEC)	me/100 g
	SA	Sand	g/100 g
	SI	Silt	g/100 g
	CL	Clay content	g/100 g
	GRAV	Rock fragment	g/100 g
	BD	Bulk density	g/cm^3
	POR	Porosity	$cm^3/100\ cm^3$

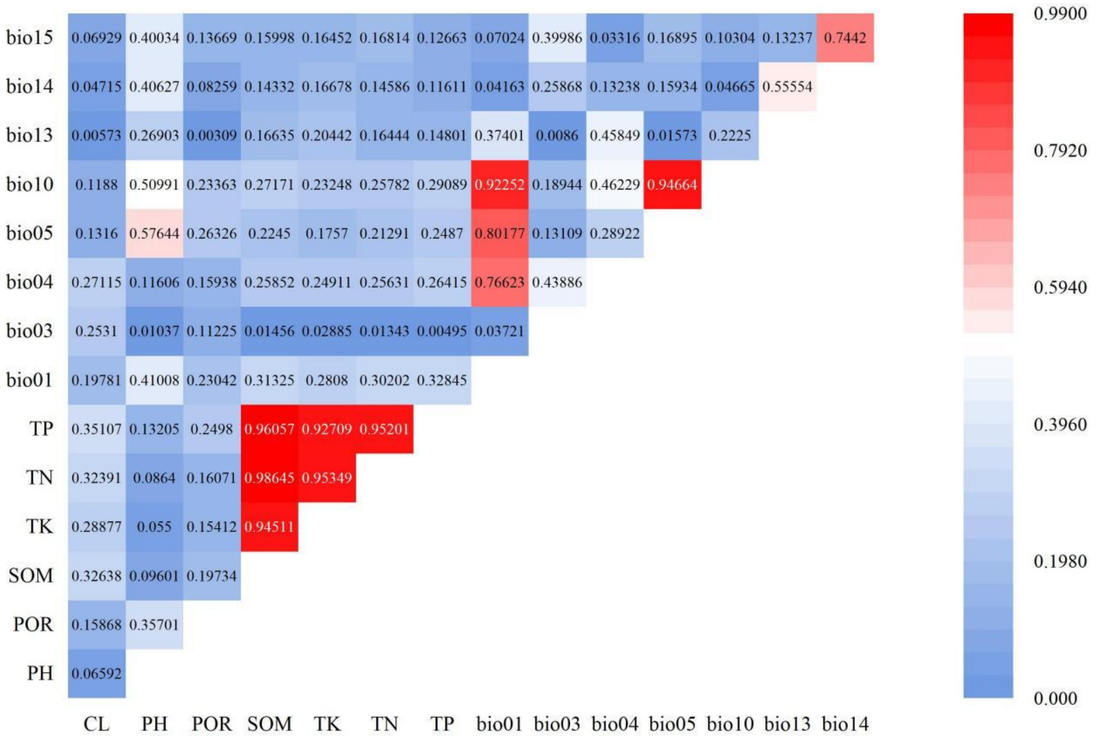

Supplementary Figure 1. Correlation analysis of environmental factors (Per cent contribution ≥ 1%).

Supplementary Table 2. Initial result of 10 MaxEnt model operations.

Variable	Percent contribution/%	Permutation importance/%
bio14	24.7	1.2
bio03	19.3	6.5
bio01	9.4	2.5
PH	9	1.4
bio15	7.4	38.5
CL	6	0.4
POR	5	2
bio13	3.8	2
TN	3.3	0
SOM	2.8	2.3
TP	2.2	2
TK	1.4	1.1
bio04	1.2	0.5
bio10	1	1.8
bio05	0.8	16.9
bio09	0.6	7.2
CEC	0.4	0.8
bio11	0.4	5.1
bio08	0.3	2.4
SI	0.2	0
bio17	0.1	0.1
SA	0.1	0.8
bio07	0.1	2.2
BD	0.1	1.2
bio19	0.1	0.1
bio16	0	0.1
bio12	0	0.4
GRAV	0	0
bio18	0	0.5
bio06	0	0
bio02	0	0

Supplementary Table 3. Weight assignment and maximum influence distance of threat factors.

THREAT	MAX_DIST	WEIGHT	DECAY
Cultivated land	1	0.6	linear
Urban land	5	1	exponential
Rural settlement	5	0.6	exponential
Other construction land	5	0.7	exponential

Supplementary Table 4. Sensitivity of different land types to threat factors.

Land use types	Habitat suitability	Cultivated land	Urban land	Rural settlement	Other construction land
Paddy field	0	0	0	0	0
Dry land	0	0	0	0	0
Forest land	0	0.8	1	0.85	0.6
Shrub land	0	0.4	0.6	0.45	0.5
Wood land	0	0.85	1	0.9	0.6
Other forestry land	0	0.9	1	0.95	0.6
High coverage grassland	1	0.4	0.6	0.45	0.5
Middle coverage grassland	1	0.45	0.65	0.5	0.55
Low coverage grassland	0.5	0.5	0.7	0.55	0.55
Canal	0	0	0	0	0
Lake	0	0	0	0	0
Reservoir pond	0	0	0	0	0
Beaches	0	0.75	1	0.8	0.5
Bench land	0	0.75	1	0.8	0.7
Urban land	0	0	0	0	0
Rural settlement	0	0	0	0	0
Other construction land	0	0	0	0	0
Sand	0	0	0	0	0
Gobi	0	0	0	0	0
Salinate field	0	0	0	0	0
Marshland	0.5	0	0	0	0
Bare land	0	0	0	0	0
Bare rock	0	0	0	0	0

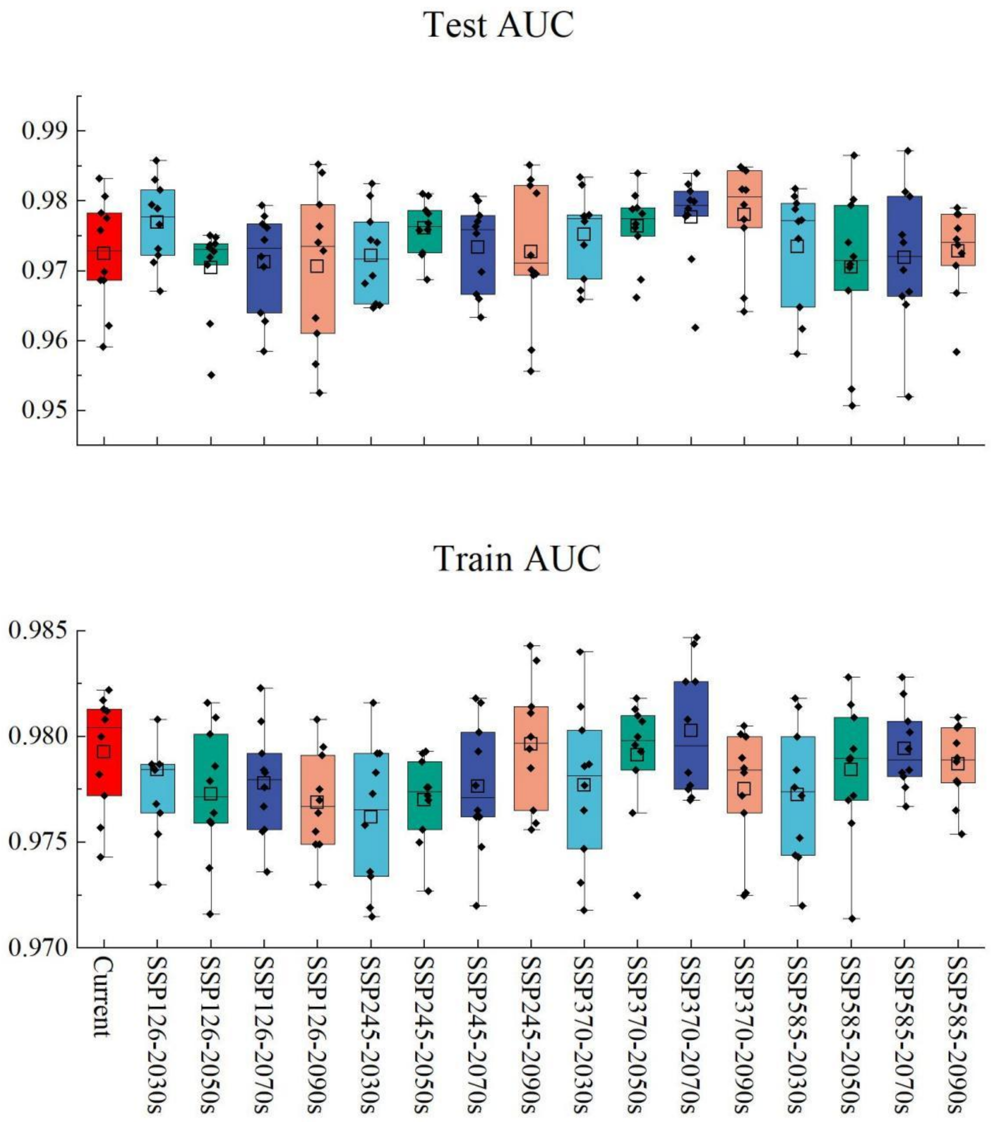

Supplementary Figure 2. Model training AUC average and test AUC average.

Supplementary Table 5. Landscape structure transfer matrix of 1980-2000.

Year	Landscape structure type	1980/km^2						
		Cropland	Forest	Grassland	Waters	Construction	Unused land	Total
2000 /km^2	Cropland	41127.99	343.03	4888.87	148.97	0.00	1385.40	47894.26
	Forest	246.96	2770.88	273.32	2.00	0.00	31.07	3324.23
	Grassland	323.76	36.59	15136.51	106.44	0.00	1043.56	16646.86
	Water	52.16	0.79	27.17	4064.4	0.00	115.74	4260.26
	Construction	164.34	1.35	27.37	0.76	3148.85	14.11	3356.79
	Unused land	162.26	0.80	1044.53	489.31	0.00	11079.45	12776.36
	Total	42077.47	3153.45	21397.77	4811.88	3148.85	13669.34	88258.76

Supplementary Table 6. Landscape structure transfer matrix of 2000-2020.

Year	Landscape structure type	2000/km^2						
		Cropland	Forest	Grassland	Waters	Construction	Unused land	Total
2020 /km^2	Cropland	39982.49	1122.74	3499.88	473.21	2155.01	2343.80	49577.13
	Forest	1237.02	1656.89	376.75	53.88	91.51	212.41	3628.47
	Grassland	2467.01	303.46	10817.02	235.44	147.69	1634.09	15604.71
	Water	372.53	39.68	175.07	1874.93	17.97	385.81	2866.00
	Construction	2180.52	65.90	266.18	52.64	841.86	230.24	3637.34
	Unused land	1654.69	135.56	1511.96	1570.15	102.74	7970.02	12945.11
	Total	47894.26	3324.23	16646.86	4260.26	3356.79	12776.36	88258.76

Supplementary Table 7. Numerical changes of forest landscape pattern indices in different decades.

Year	NP	PD	MPS	FRAC_AM	DIVISION	AI	Landscape fragmentation index
1980	2450	0.0277	874.1633	1.1358	0.998	57.9582	0.3443
2000	2296	0.026	725.2178	1.1227	0.9988	54.8165	0.3589
2020	2334	0.0264	669.1088	1.1211	0.9989	53.5405	0.3639

Supplementary Table 8. Suitable area of *G. manshurica* proportion of different degrees of fragmentation.

Type	1980		2000		2020	
	Area/km^2	Proportion/%	Area/km^2	Proportion/%	Area/km^2	Proportion/%
Extremely low fragmentation	6979	28.30	4845	24.03	4268	22.32
Low fragmentation	7558	30.65	5975	29.63	7512	39.29
Mid fragmentation	6646	26.95	6182	30.66	4215	22.05
High fragmentation	1712	6.94	1525	7.56	1398	7.31
Extremely high fragmentation	1763	7.15	1638	8.12	1725	9.02
Total area	24658	100	20165	100	19118	100

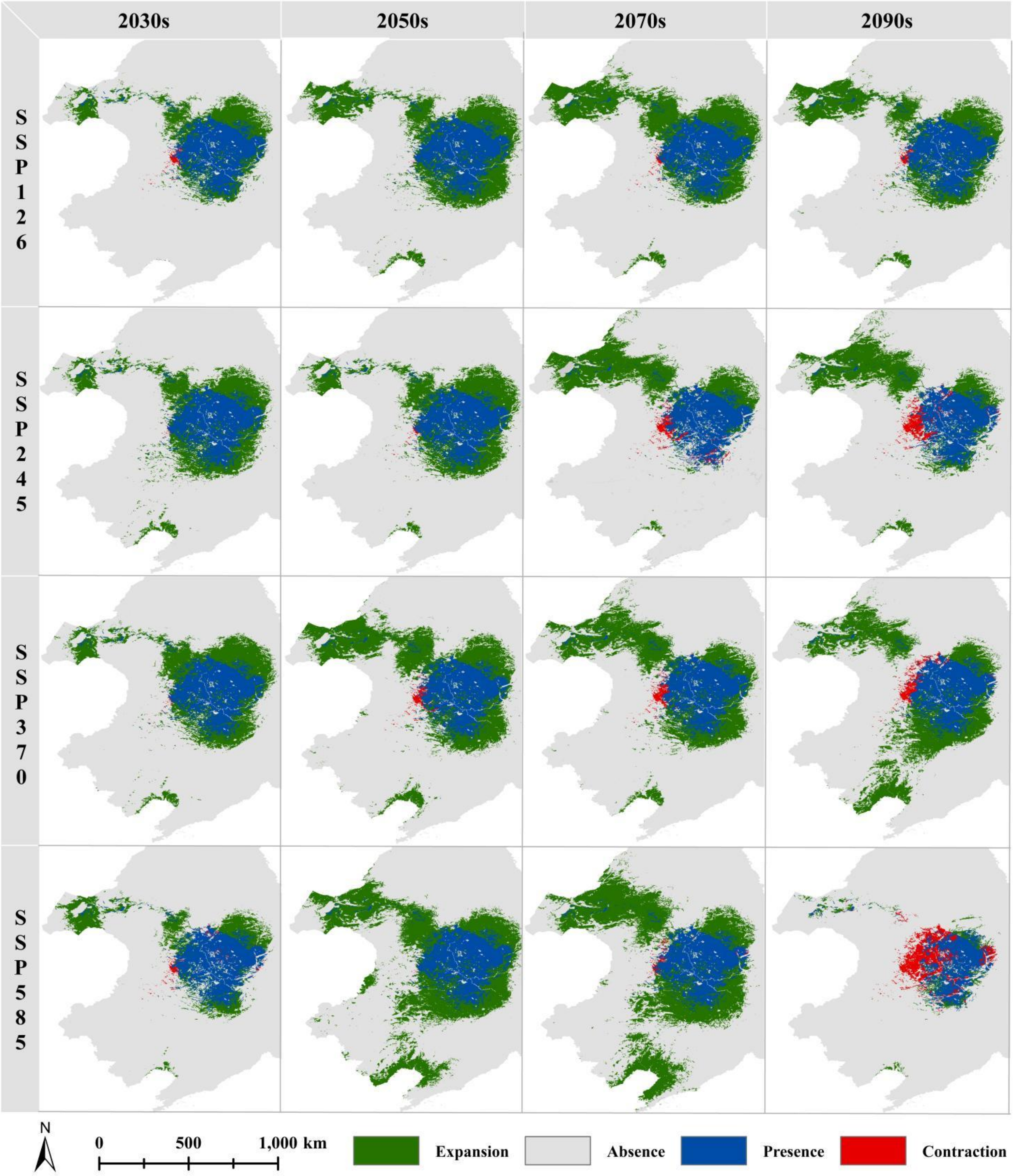

Supplementary Figure 3. Distribution change of FPDs of *G. manshurica* in different SSPs and year.

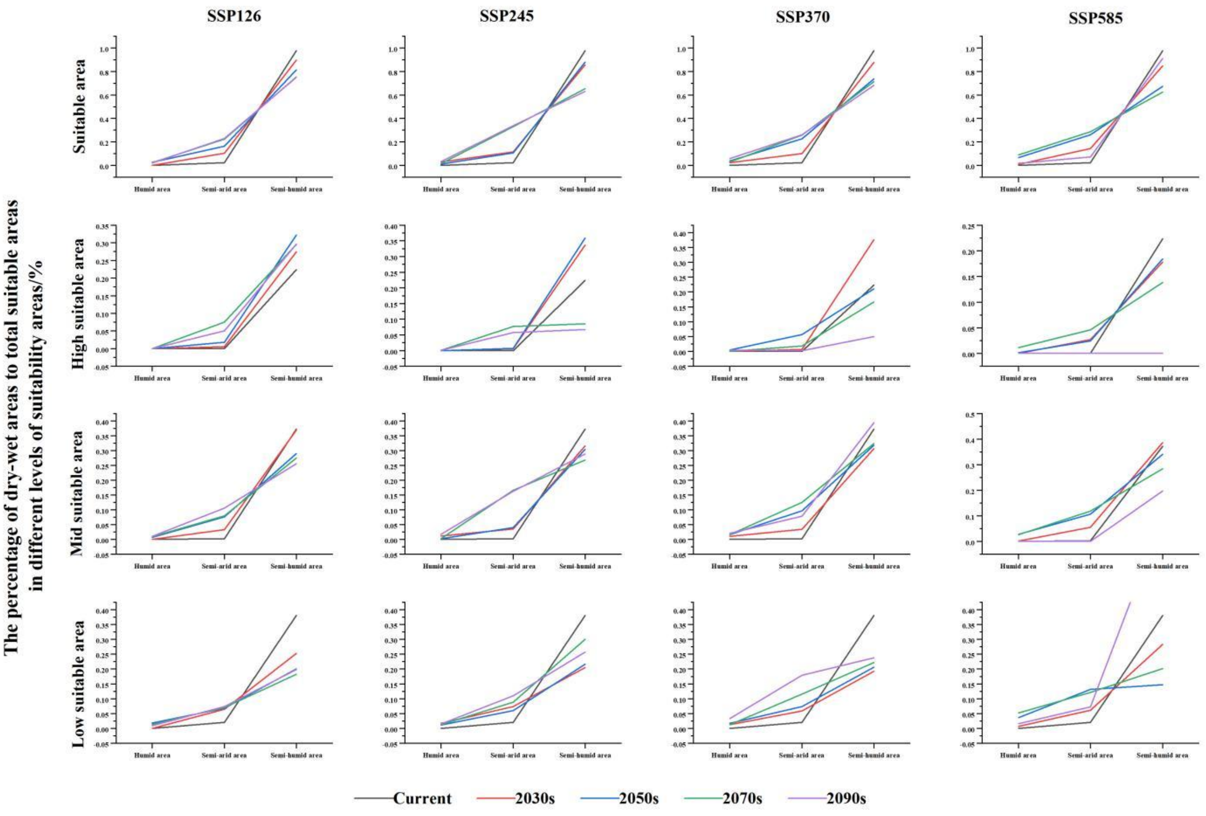

Supplementary Figure 4. Area of dry and wet area in each suitable grade of *G. manshurica.*

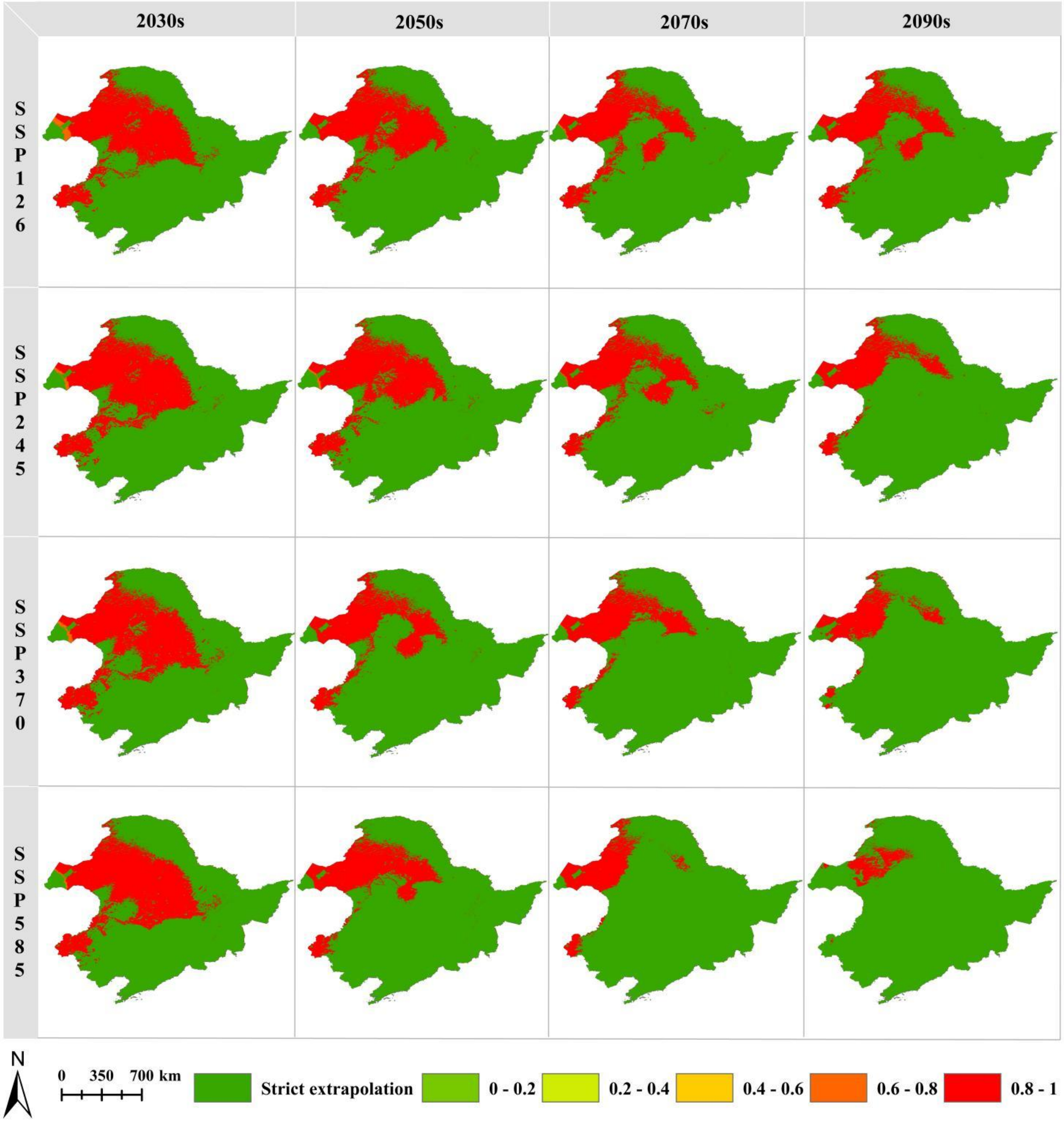

Supplementary Figure 5. The results of the Mobility-Oriented Parity (MOP) analysis.

Supplementary Table 9. Environmental variables and their contributions and suitable ranges.

Variable	Percent contribution/%	Permutation importance/%
bio15	38.1	57.7
bio03	20.4	26.8
bio01	17.3	7.7
CL	13.1	1.3
PH	4.9	1.1
POR	2.5	0.7
TN	1.7	1.2
bio13	1.6	1.6
bio14	0.4	1.9

Supplementary Table 10. Climate factors in different climate scenario combination and current climate conditions t-test results

Climate scenario		bio01	bio03	bio15
2030s	SSP126	< 2.2e-16**	0.9481	< 2.2e-16**
	SSP245	< 2.2e-16**	0.5923	< 2.2e-16**
	SSP370	< 2.2e-16**	0.02505*	< 2.2e-16**
	SSP585	< 2.2e-16**	0.09222	< 2.2e-16**
2050s	SSP126	< 2.2e-16**	0.4069	< 2.2e-16**
	SSP245	< 2.2e-16**	0.5393	< 2.2e-16**
	SSP370	< 2.2e-16**	0.0004196**	< 2.2e-16**
	SSP585	< 2.2e-16**	0.1548	< 2.2e-16**
2070s	SSP126	< 2.2e-16**	0.004976**	< 2.2e-16**
	SSP245	< 2.2e-16**	0.1866	< 2.2e-16**
	SSP370	< 2.2e-16**	0.6797	< 2.2e-16**
	SSP585	< 2.2e-16**	0.001461**	< 2.2e-16**
2090s	SSP126	< 2.2e-16**	0.7088	< 2.2e-16**
	SSP245	< 2.2e-16**	0.01426	< 2.2e-16**
	SSP370	< 2.2e-16**	<2.2e-16**	< 2.2e-16**
	SSP585	< 2.2e-16**	<2.2e-16**	< 2.2e-16**

Note: * shows significant difference at 0.05 level, and **shows significant difference at 0.01 level.

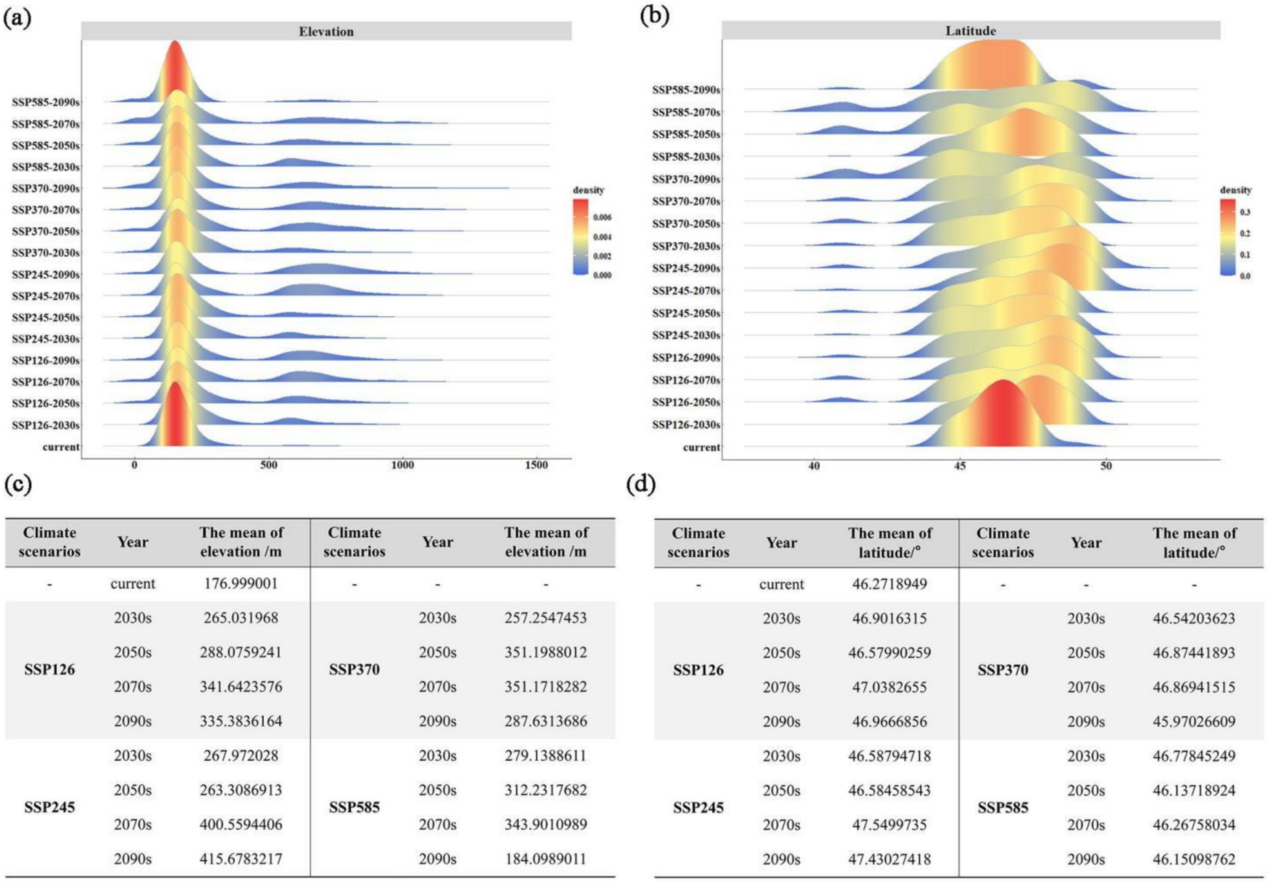

(c)

Climate scenarios	Year	The mean of elevation /m	Climate scenarios	Year	The mean of elevation /m
-	current	176.999001	-	-	-
**SSP126**	2030s	265.031968	**SSP370**	2030s	257.2547453
	2050s	288.0759241		2050s	351.1988012
	2070s	341.6423576		2070s	351.1718282
	2090s	335.3836164		2090s	287.6313686
**SSP245**	2030s	267.972028	**SSP585**	2030s	279.1388611
	2050s	263.3086913		2050s	312.2317682
	2070s	400.5594406		2070s	343.9010989
	2090s	415.6783217		2090s	184.0989011

(d)

Climate scenarios	Year	The mean of latitude/°	Climate scenarios	Year	The mean of latitude/°
-	current	46.2718949	-	-	-
**SSP126**	2030s	46.9016315	**SSP370**	2030s	46.54203623
	2050s	46.57990259		2050s	46.87441893
	2070s	47.0382655		2070s	46.86941515
	2090s	46.9666856		2090s	45.97026609
**SSP245**	2030s	46.58794718	**SSP585**	2030s	46.77845249
	2050s	46.58458543		2050s	46.13718924
	2070s	47.5499735		2070s	46.26758034
	2090s	47.43027418		2090s	46.15098762

Supplementary Figure 6. Elevation and latitude changes within the FPDs of *G. manshurica* (a, c. elevation; b, d. latitude).

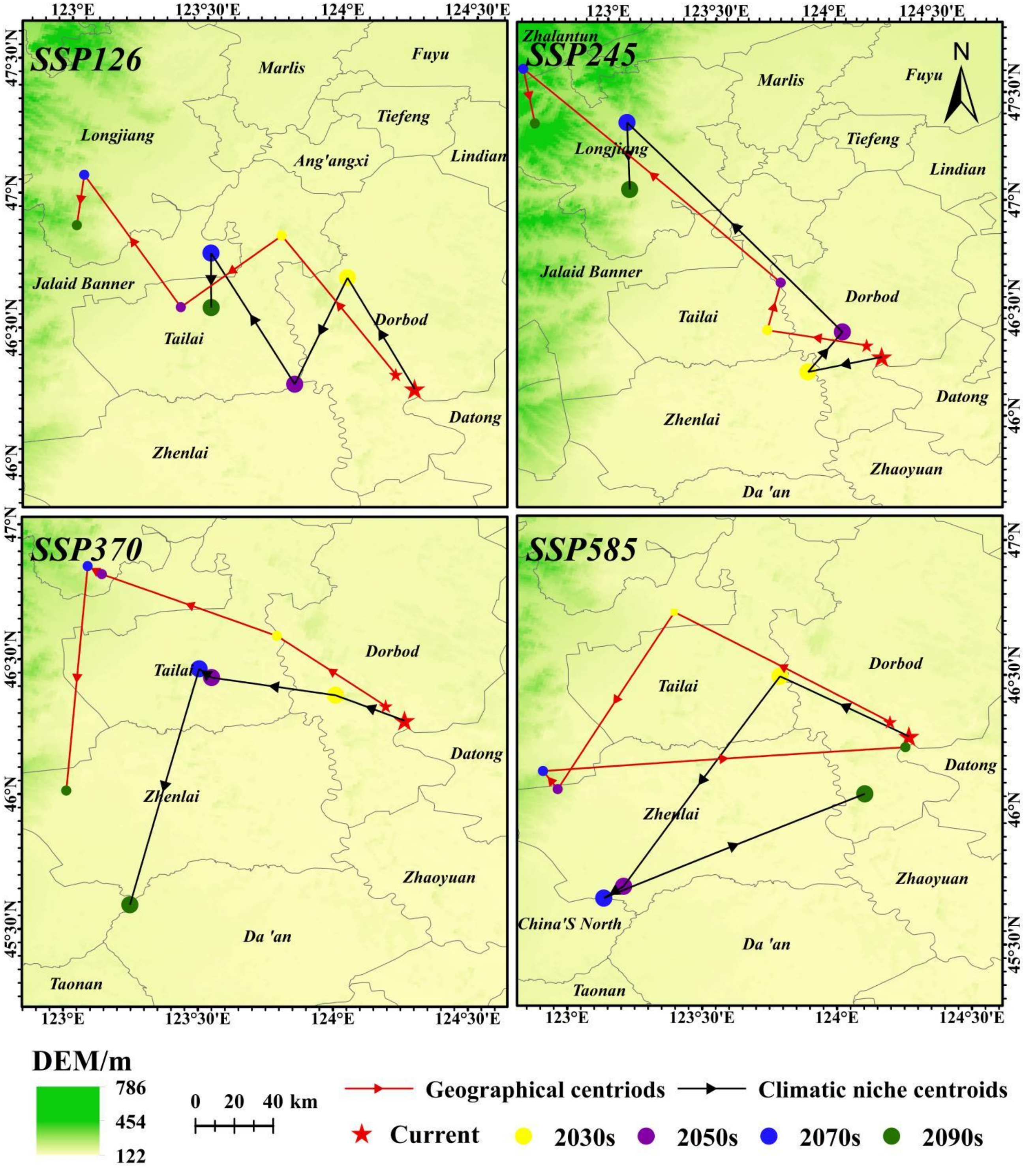

Supplementary Figure 7. Comparison of the geographical centroid and climate niche centroid.
